# Electrospun Poly(carbonate-urea-urethane)s Nonwovens with Shape-Memory Properties as a Potential Biomaterial

**DOI:** 10.1021/acsbiomaterials.3c01214

**Published:** 2023-11-30

**Authors:** Karolina Rolińska, Hadi Bakhshi, Maria Balk, Anna Blocki, Amit Panwar, Michał Puchalski, Michał Wojasiński, Magdalena Mazurek-Budzyńska

**Affiliations:** † Faculty of Chemistry, Warsaw University of Technology, Noakowskiego 3, 00-664 Warsaw, Poland; ‡ Faculty of Chemistry, University of Warsaw, Pasteura 1, 02-093 Warsaw, Poland; § Department of Life Science and Bioprocesses, Fraunhofer Institute for Applied Polymer Research IAP, Geiselbergstraße 69, 14476 Potsdam, Germany; ∥ Institute of Active Polymers, Helmholtz-Zentrum Hereon, Kantstraße 55, 14513 Teltow, Germany; ⊥ Institute for Tissue Engineering and Regenerative Medicine, The Chinese University of Hong Kong, Shatin, New Territories 999077, Hong Kong; # School of Biomedical Sciences, Faculty of Medicine, The Chinese University of Hong Kong, Shatin, New Territories 999077, Hong Kong; ∇ Center for Neuromusculoskeletal Restorative Medicine, The Chinese University of Hong Kong, Shatin, New Territories 999077, Hong Kong; ○ Institute of Material Science of Textiles and Polymer Composites, Faculty of Material Technologies and Textile Design, Lodz University of Technology, ul. Żeromskiego 116, 90-924 Łódź, Poland; ◆ Faculty of Chemical and Process Engineering, Department of Biotechnology and Bioprocess Engineering, Laboratory of Biomedical Engineering, Warsaw University of Technology, Waryńskiego 1, 00-645 Warsaw, Poland

**Keywords:** poly(carbonate-urea-urethane)s, electrospinning, shape-memory effect, biomaterial

## Abstract

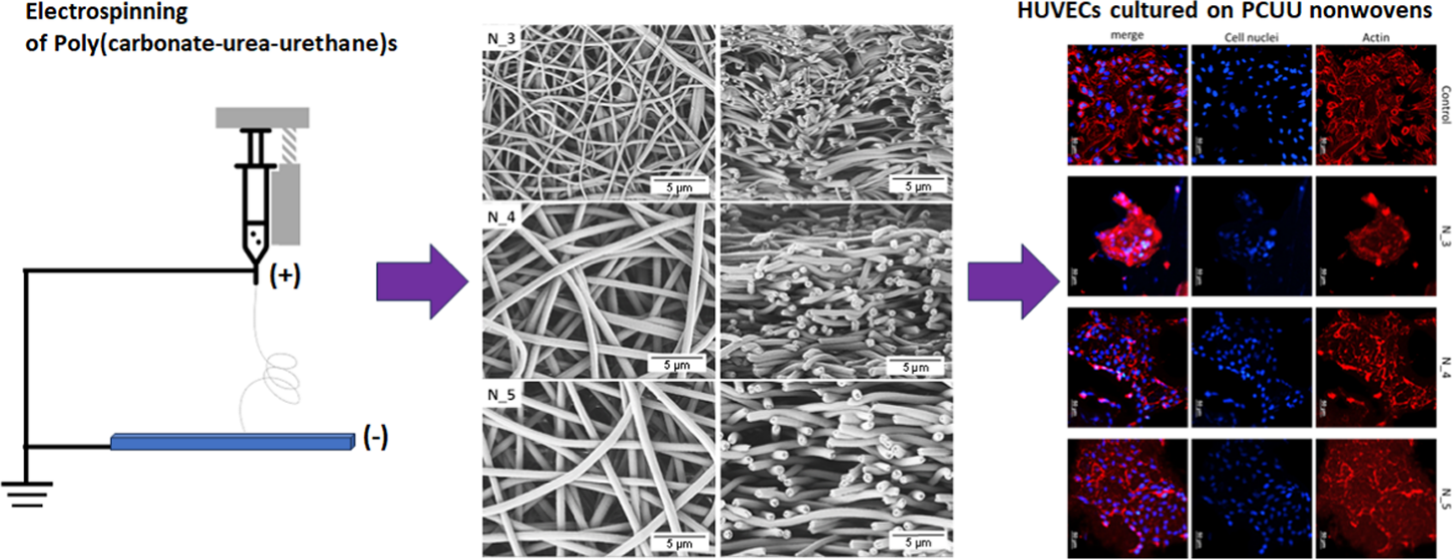

Poly(carbonate-urea-urethane) (PCUU)-based scaffolds exhibit various desirable properties for tissue engineering applications. This study thus aimed to investigate the suitability of PCUU as polymers for the manufacturing of nonwoven mats by electrospinning, able to closely mimic the fibrous structure of the extracellular matrix. PCUU nonwovens of fiber diameters ranging from 0.28 ± 0.07 to 0.82 ± 0.12 μm were obtained with an average surface porosity of around 50–60%. Depending on the collector type and solution concentration, a broad range of tensile strengths (in the range of 0.3–9.6 MPa), elongation at break (90–290%), and Young’s modulus (5.7–26.7 MPa) at room temperature of the nonwovens could be obtained. Furthermore, samples collected on the plate collector showed a shape-memory effect with a shape-recovery ratio (*R*_r_) of around 99% and a shape-fixity ratio (*R*_f_) of around 96%. Biological evaluation validated the inertness, stability, and lack of cytotoxicity of PCUU nonwovens obtained on the plate collector. The ability of mesenchymal stem cells (MSCs) and endothelial cells (HUVECs) to attach, elongate, and grow on the surface of the nonwovens suggests that the manufactured nonwovens are suitable scaffolds for tissue engineering applications.

## 1 Introduction

The high demand and popularity of polyurethanes (PUs) are brought about by their unique physical and mechanical properties, such as durability, flexibility, and abrasion resistance. They are used for the production of flexible and rigid foams, adhesives, gaskets, and coatings with high-performance parameters.^1−3^ Due to their biostability and biocompatible properties with certain tissues, polyurethanes have also been used as biomedical materials.^4−10^ An interesting group of materials among polyurethanes is poly(carbonate-urethane)s (PCUs). Aliphatic polycarbonates constitute a group of polymers that are much more resistant to hydrolytic and enzymatic degradation than polyesters and toward oxidative conditions in comparison to polyethers and have been thus employed in various biomedical devices and applications.^11−14^ PCUs have gained much attention in the biomedical field as good competitors to poly(ester-urethane)s^15^ and poly(ether-urethane)s^16^ as they combine good environmental resistance with a variety of mechanical and thermal properties of polyurethanes.

The growing demand for organs and tissues for transplantation is one of the greatest challenges in modern medicine. Tissue engineering, a dynamically developing field, is a biomedical research area that attempts to address this demand. Scaffolds used in tissue engineering should exhibit biocompatibility with the tissue to be engineered and maintain appropriate mechanical properties in the complex environment of the body. Such scaffolds enable the restoration or regeneration of the tissue, e.g., bone, cartilage, vascular, nervous, skeletal muscle or skin, by acting as a matrix, which allows cell infiltration, proliferation, and thus integration with the surrounding tissues.^17−95^ Various technologies exist that allow the adjustment of the properties of the resulting scaffolds and the controlled and targeted release of bioactive agents.

An important aspect of tissue engineering scaffolds is their ability to closely mimic the structure of the fibrous extracellular matrix so that the basic goals of cell organization, survival, and function can be met. One method that enables the creation of fibrous structures similar to the natural extracellular matrix is electrospinning (ES).^19^ The application of the technique of manufacturing fibers by ES compared to traditional spinning methods is distinguished by the smaller diameter of the obtained fibers. ES allows to obtain uniform fibers with diameters from about 100 nm to about 1 μm,^20^ similar to the structure of the natural extracellular matrix.^21,22^ Therefore, they show a high potential for creating artificial functional tissues.^23^ Scaffolds made of polymer-based electrospun nanofibers can also successfully deliver therapeutic agents, genetic material, and tools for genome editing.^24^ ES materials are often used as dressing materials due to their good barrier properties and oxygen permeability.^25−28^ Polyurethanes are often chosen as a substrate to produce nanofibers due to their chemical stability, good mechanical properties, and excellent fiber-forming properties, enabling the formation of nanofibers.^27^ Electrospun PU nonwovens have been applied in many potential applications, e.g., in high-efficiency air filters, protective textiles, dressing materials, sensors, and drug carriers.^26,28−97^ PCU fibers can be obtained by using the ES technique^30,31^ to be potentially used in tissue engineering.^32^ Qiu et al. produced PCU vascular grafts (VGs) by ES closely mimicking the native blood vessel structure and mechanical strength.^33^ The fact that VGs produced by ES from PCU^34^ and poly(urea-urethane)^35^ elastomers are already available in the market demonstrates the high potential of VGs of this type. However, these VGs are recommended for hemodialysis vascular access only, which indirectly suggests the need to fit their mechanical properties and biological effects to a wider variety of applications. In other studies, Bergmeister et al. have shown that compared to PTFE, PCU grafts showed transmural ingrowth of vascular-specific cells, resulting in a structured neovessel formation around the graft.^36^ Hard block degradable PCUs were synthesized with the usage of poly(hexamethylene carbonate) as soft blocks, hexamethylene diisocyanate as hard blocks, and bis(3-hydroxypropylene)carbonate as a carbonate chain extender.^37^ Ehramann et al. concluded that a lower concentration of hard block was more suitable for vascular grafts, with slight signs of inflammatory reactions.^37^ Recently, a polyhedral oligomeric silsesquioxane PCUU graft fabricated by an electrospray technique with phase inversion was implanted into the carotid artery of 12 sheep for 9 months.^38^ These grafts did not display any hyperplasia of the intima, calcification, or aneurysms of anastomoses and showed performance characteristics comparable to those of native arteries. The patency rate of VGs was 64%. Li and co-workers fabricated heparin-grafted electrospinning PCU artificial vascular scaffolds in another study. The final product showed excellent biocompatibility for this application in in vitro and in vivo studies with a low-rate thrombus formation and hemolysis.^39^ Furthermore, a green chemistry approach was utilized to obtain high-molecular-weight nonisocyanate polyurethane (NIPU) PCUs obtained through transurethanization of 1,6-hexanedicarbamate with polycarbonate diols, which can be processed by ES. Both fibroblasts and epithelial cells displayed good adhesion and proliferation on the electrospun NIPU mats.^40^ More recently, a polyhedral oligomeric silsesquioxane poly(carbonate-urea-urethane) (POSS–PCU) was obtained and for the first time used in human studies as a bypass graft or synthetic trachea;^47^ it did not show cytotoxic effects and allowed antiplatelet and anticoagulant function, allowing endothelial cells to grow.^48^ Several modifications of POSS–PCU have been reported, especially for enhancing the capture of endothelial progenitor cells,^49^ enhancing the response and osteogenic differentiation of adipose-derived stem cells,^50^ or for improving the surface for the immobilization of various ECM proteins.^51^ Recently, POSS–PCU were utilized in the electrospinning process and provided appropriate surface and mechanical properties for the fabrication of small-diameter vascular grafts with a single-layer endothelial barrier at the luminal surface.^52^

Furthermore, the development of poly(carbonate-urea-urethane) (PCUU) called MyoLink obtained based on MDI, poly(carbonate)diol, and ethylene diamine was reported to have optimal properties for human umbilical vein endothelial cell (HUVEC) adhesion^41^ and increased chemical resistance in in vivo studies when compared to poly(ether-urethane) graft (Pulse-Tec).^42^ Also, a long-term in vivo study (18–36 months) on dogs implanted with graft in the aorta-iliac position showed no infection or inflammation in the surrounding tissue.^43^ VG prepared based on the same material was functionalized on the luminal surface with heparin and arginine-glycine-aspartate peptide, which were chosen as anticoagulant and cell attachment moieties. No change of mechanical properties was reported, and the moieties were attached uniformly.^44^ Furtermore, functionalization accelerated and improved endothelial cell retentions were observed.^45^ Coating of MyoLink with fibronectin-like engineered polymer protein-plus enhanced smooth muscle cell attachment.^46^ To our best knowledge, this is the only published report concerning PCUU-based materials for medical applications.

In addition, attention should be paid to intelligent materials, which can change their properties in response to an external stimulus. An example is the shape-memory polymer (SMP), which, after a programming process and under an appropriate external stimulus, can change its shape from a temporary to a permanent form.^53^ Shape-memory polyurethanes (SMPUs) are the best-known types of SMPs for biomedical applications due to their biocompatibility with certain tissues, biodegradability, easy structure manipulation, and transition temperature close to the body temperature.^54^ SMPUs made it possible to change the shape and architecture of the internal matrix while maintaining the viability of the cells placed on it. Tseng et al. have shown that these scaffolds can be used to control the alignment of the cell actin filaments and nuclei. In addition, they demonstrated that the use of the shape-shifting process and scaffold architecture can influence the morphological behavior of cells.^55^ Shape-memory core–shell nanofibers were obtained by coaxial ES. The use of this method enabled the development of a new technique for encapsulating drugs and therapeutic agents. ES fibers were produced based on the SMPU solution based on polycaprolactone as the core and the coating solution—pyridine polyurethane with shape memory capability. In addition to excellent SME, the core–sheath nanofibers also showed excellent antibacterial activity against both Gram-negative and Gram-positive bacteria.^56^ The usefulness of SMP for the development of self-adjusting tissue engineering implants and a local drug delivery system has been described in the example of poly(ester-urethanes),^4,57^ copolyesters,^58,59^ or poly(ester-carbonates).^60^ Henderson and co-workers have recently demonstrated the enzymatic triggering of shape-memory PCL-Pellethane in direct response to the presence of enzyme-secreting human cells.^61^

Based on these findings, we found it crucial to continue studies on the potential usage of PCUU in the field of tissue engineering. For the purposes of the project, we selected PCUU materials exhibiting SME,^15,62,96^ which are known for good hydrolytic stability, and used them as substrates in electrospinning of nanofibers. We investigated the effect of the ES parameters on the nonwoven morphology and the thermal and mechanical parameters. Then, we performed biological tests using mesenchymal stem cells (MSCs) and human umbilical vein endothelial cells (HUVECs). Furthermore, the one-way shape-memory effect of the selected nonwovens was investigated.

## 2 Experimental Section

### 2.1 Materials

1,10-Decanediol (purity 98%) and isophorone diisocyanate (IPDI) (purity 98%) were purchased from Sigma-Aldrich (Poznan, Poland). Potassium carbonate (K_2_CO_3_) (purity ≥ 99%), 1,4-dioxane (purity 99%), dimethyl carbonate (DMC) (purity 99%), chloroform (purity ≥ 99%), tetrahydrofuran (THF) (purity ≥ 99%), and *N,N*-dimethylformamide (DMF) (purity ≥ 99%) were purchased from POCH (Gliwice, Poland). Materials were used without any further purification. Oligo(decamethylene carbonate)diol (OCD) and PCUU were synthesized according to the literature (Scheme S1, Supporting Information).^62^ The amounts of reagents used are shown in Table S1 in the Supporting Information. The structure confirmation of intermediate products was performed. Figures S1–S3 in the Supporting Information present ^1^H NMR spectra, and Figure S4 shows Fourier transform infrared (FTIR) spectra. ^1^H NMR spectra of PCUU electrospun nonwovens are presented in Figure S5 in the Supporting Information. FTIR spectra of PCUUs are shown in Figures S6–S8 in the Supporting Information.

### 2.2 ES Solution Preparation and Process Parameters

PCUU was dissolved in a DMF/THF mixture (50/50, w/w) at room temperature by magnetic stirring for 72 h. Solutions of 3, 4, and 5 wt % were prepared. The ES process was performed in the laboratory spinning unit equipped with a plate collector (7 × 7 cm^2^) or drum collector (MTI Corporation, model: MSK-ESDC-80–4000) with dimensions of 80 × 200 mm^2^ with a speed controller. The rotational speed of the drum collector was 50 or 150 rpm. The distance between the plate and drum collectors and the nozzle was 20 cm. Each solution was placed in a 20 cm^3^ syringe and electrospun on the plate/drum collector (covered with aluminum foil) through a 22 G needle with a 0.41 mm inner diameter. The power supply was set up for a positive voltage of 18 kV. The flow rate of the solution was set up on the syringe pump at 1.5 cm^3^·h^–1^. The relative humidity (RH) and temperature values at the time of the experiments ranged from 40 to 52% and 24 to 27 °C. Nonwovens were named accordingly: **N**_**X**_**Y**, where **X** means the concentration of the solution used for the ES process (wt %) and **Y** is the rotation speed of the collector in rpm (if the rotatory collector was applied). For example, **N_3_50** means that the electrospun mat was obtained using a 3 wt % solution of PCUU on the rotatory collector with a speed of 50 rpm. In the case of the plate collector, the names were given accordingly: **N_X**.

### 2.3 Instruments and Methods

^1^H NMR and spectra were recorded at 298 K on a Varian VXR 400 MHz spectrometer (Palo Alto, California) using tetramethylsilane as an internal reference and CDCl_3_ as a solvent and were analyzed with MestReNova v.6.2.0–7238 (Mestrelab Research S.L) software. The error of the method was estimated based on the error of the integral peak area (around 5%).

Attenuated total reflectance (ATR)–FTIR spectra were recorded on a Thermo Scientific Nicolet iS5 FTIR spectrometer using an ATR iD7 accessory; 32 scans were recorded for each sample.

The kinematic viscosity (ν) and density of solutions prepared by dissolving PCUU in DMF/THF (1:1 wt %) were analyzed with an automatic Ubbelohde capillary viscosimeter (PSV1, Lauda, Lauda-Königshofen, Germany) in a temperature-controlled water bath (Lauda E 200, Lauda-Königshofen, Germany) at 23 °C. The corresponding dynamic viscosity η was calculated according to eq 1
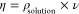
1

The front view of samples was investigated by scanning electron microscopy (SEM) measurements, where samples were cut using sharp razor blades and stuck on specific holders with conductive adhesive. The samples were sputtered with gold, achieving a thickness of 5 nm. Samples were then investigated with a desktop-SEM Phenom G2 instrument from PhenomWorld (LOT-Oriel Group Europe, Darmstadt, Germany). To investigate the cross section, samples were moistened with 2-propanol and cooled with liquid nitrogen. Samples were broken by means of a blade and stuck on specific holders with conductive adhesive. The samples were sputtered with iridium, achieving a thickness of 4 nm, and investigated with a SEM Supra 40VP (Carl Zeiss Company, Oberkochen, Germany).

The surface porosity of the electrospun samples was calculated from SEM images of the top sides at a magnification of 5000 using ImageJ software (version 1.52p). For this purpose, the SEM images were converted into 8-bit binary (black and white) images and underwent thresholding, where the black areas represent pores and the white areas represent nanofibers. This provided a clear separation between pores and nanofibers. The binary image was then analyzed using the “analyze particles” tool built into ImageJ software.

Differential scanning calorimetry (DSC) was performed on a Netzsch DSC 204 (Netzsch Ltd. Selb. Germany) in sealed Al pans under a N_2_ atmosphere between −70 and 150 °C with heating and cooling rates of 10 °C·min^–1^.

Dynamic mechanical thermal analysis (DMTA) measurements were performed on an Eplexor 25 N (Gabo. Ahlden. Germany) equipped with a 25 N load cell using the standard-type test specimen (DIN EN ISO 527-2/1BB). The applied oscillation frequency was 1 Hz. The measurements were performed in the temperature sweep mode from −100 to 150 °C with a constant heating rate of 3 °C·min^–1^.

Wide-angle X-ray scattering (WAXS) measurements were conducted at ambient temperature in transmission geometry utilizing the X-ray diffraction system Bruker D8 Discover (generator operated at 40 kV and 40 mA) with a two-dimensional detector from Bruker AXS (Karlsruhe, Germany). The X-ray beam (Cu Kα1-radiation λ = 0.154 nm) was provided by a graphite monochromator and a pinhole collimator with an opening of 0.8 mm. The sample-to-detector distance was 15 cm applying an irradiation time of 120 s. Integration of the two-dimensional intensities gave linear intensity curves *I* (2θ). Crystallinity values were calculated as an average of three individual fits of the scattering curve with Pearson 7 functions utilizing TOPAS (R) software from Bruker AXS.

Tensile tests were conducted with standard samples (ISO 527-2/1BB) cut from mats on a tensile tester Z75 (Zwick, Ulm, Germany) equipped with a thermochamber (Mytron Bio- and Solartechnik, Heilbad Heiligenstadt, Germany), a temperature controller Eurotherm control 2408 (Eurotherm Regler, Limburg, Germany), and load cells suitable to determine maximum forces of 200 N (Zwick, Ulm, Germany). The strain rate in the uniaxial tensile test was 10 mm min^–1^. The average value of the tensile strength (σ), elongation at break (ε), and Young’s modulus (*E*) for each type of material was determined from five specimens. Measurements were performed at room temperature and 37 °C.

Shape-memory effect (SME) was measured using a DMTA Q800 machine (TA Instruments, New Castle, Delaware). Five consecutive cycles consisting of heating, stress loading, relaxation, cooling, stress unloading, relaxation, and heating were conducted. Shape-memory performance was evaluated by the ability of SMPs to fix the temporarily deformed shape (shape-fixity ratio, *R*_f_) and the ability to recover the permanent shape (shape-recovery ratio, *R*_r_).^63^ The first recorded cycle was not included in the SME analysis since it has been considered an annealing cycle. The *R*_f_ and *R*_r_ were calculated according to eqs 2 and 3. The nonwoven sample with dimensions of ca. 10 × 11 × 0.02 mm^3^ was deformed to ε_prog_ at *T*_prog_ = 50 °C with the elongation speed of 10 mm min^–1^. The sample was then left for 10 min at ε_prog_ to allow relaxation, while the force applied to the samples was equal to 0.5 N. The sample was then cooled to *T*_low_ = −5 °C with a cooling rate of 10 °C min^–1^, while it elongated to ε_l_ due to elongation-induced crystallization. After unloading the stress (σ_0_ = 20 mN), and an equilibration time of 10 min, the temporary shape ε_u_ was fixed. Then, the sample was heated to *T*_prog_ with a heating rate of 10 °C min^–1^ and kept at this temperature for 30 min, resulting in shape recovery to ε_p_. Afterward, the next cycle was performed. Based on DSC results (Table S3), the values of *T*_low_ and *T*_prog_ were chosen. *T*_low_ was chosen as *T*_c_ because at this temperature crystallization of the sample is the fastest. *T*_prog_ was chosen as the temperature, in which the crystalline phase of the sample was fully molten. Therefore, we set *T*_prog_ to be 50 °C as it is above *T*_m_ of the samples.
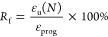
2
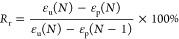
3

#### 2.3.1 Biological Tests

##### 2.3.1.1 Cell Culture

Human umbilical vein endothelial cells (HUVECs; cat. #PCS-100-013; ATCC) at passage 4 were thawed and cultured in endothelial cell growth medium-2 (EGM-2, Lonza, cat. # CC-3162). Human bone marrow mesenchymal stem cells (Human BM MSCs; cat. # 3092430; Millipore Temecula, California) at passage 4 were thawed and cultured in low-glucose Dulbecco’s modified Eagle’s medium (DMEM, cat. #31600092; Gibco, Life Technologies, Grand Island, New York) supplemented with 10% fetal bovine serum (FBS, cat. #16000044; Gibco, Life Technologies, Grand Island, New York) and 1% penicillin–streptomycin (P/S, cat. #15140-122; Gibco, Life Technologies, Grand Island, New York). The subculture included trypsinization with TrypLE Express enzyme (cat. #12605-010; Gibco, Life Technologies, Grand Island, New York) and centrifugation at 25 °C for 5 min with 300 relative centrifugal force (RCF).

##### 2.3.1.2 Cytotoxicity Studies

Selected PCUU nonwovens were cut into squares (5 × 5 mm^2^) and suspended in 70% ethanol for 24 h for disinfection. Later, scaffolds were transferred to sterile Petri dishes for air-drying in a laminar hood for overnight drying under sterile conditions. After disinfection, selected PCUU nonwovens (5 × 5 mm^2^) were suspended in a 1 cm^3^ DMEM/EGM-2 medium in a centrifuge tube (1.5 cm^3^) for 48 h at 37 °C in a CO_2_ incubator to obtain FC^d^ (film-conditioned DMEM medium) and FC^e^ (film-conditioned EGM-2 medium) and stored at 4 °C for future use. Further, FC^d^ and FC^e^ mediums were used to study the effect of the conditioned medium on MSCs and HUVECs, respectively, for indirect cytotoxicity studies. MSCs/HUVECs at passage 5 (P5) were seeded at ∼2500 cells/well of a 96-well plate with 150 μL/well DMEM/EGM-2 media and incubated for 24 h in a CO_2_ incubator. After 24 h, the medium was replaced with their designated film-conditioned medium **(**FC^d^ and FC^e^) and incubated for 72 h. Cell proliferation rates were analyzed to study the cytotoxicity of the nonwovens using cell counting kit-8 (CCK-8) (APExBIO, Houston, Texas, Cat. #K1018). As per kit instructions, 10 μL of CCK-8 solution in 100 μL of DMEM/EGM-2 medium was added to each well after aspirating the old medium and incubated for 3 h at 37 °C. For quantification, absorbance at 450 nm was measured using a microplate reader (Thermo Scientific, Multiskan GO).

##### 2.3.1.3 Cell Seeding on PCUU Nonwovens

Selected PCUU nonwovens were cut into squares (2.5 × 2.5 cm^2^) and suspended in 70% ethanol for 24 h for disinfection. Later, scaffolds were transferred to sterile Petri dishes for air-drying in a laminar hood for overnight drying under sterile conditions. After disinfection, nonwovens were inserted in the wells of a 48-well plate and washed with 1× PBS three times. MSCs (P5)/HUVECs (P5) were seeded on PCUU nonwovens at 8000/well with a 150 μL/well DMEM/EGM-2 medium and incubated for 24 h. After 24 h, 150 μL of DMEM/EGM-2 medium was added to the wells and incubated for another 24 h. Further, the cells were analyzed for cell viability and attachment by cytochemistry.

##### 2.3.1.4 Cell Viability

For cell viability, a live/dead cell assay for the cells seeded on PCUU nonwovens was carried out after 48 h of cell culture. The assay was performed using calcein AM (2 μM, C1430 Life technologies) and ethidium homodimer-1, EthD-1 (E1169, 4 μM, Life Technologies), as per the manufacturer’s instructions. A master mixed staining solution was prepared in 1× PBS with calcein (1:2000) and EthD-1 (1:500). 400 μL of master mix staining solution was added to the wells containing PCUU nonwovens with HUVECs or MSCs after washing twice with 1× PBS and incubated for 15 min after wrapping with aluminum foil. Further, the staining solution in the wells was replaced with fresh 1× PBS and removed from the wells, and stained cells were observed under a fluorescent microscope (Olympus IX83; Olympus, Tokyo, Japan). After imaging, live and dead cells were counted using ImageJ software. All of the measurements were carried out in triplicates.

Statistical analysis was performed with the one-way analysis of variance algorithm. Summarized results from at least three independent in vitro biological runs are presented; three replicates each were compared using post hoc Tukey’s tests.

##### 2.3.1.5 Cytochemistry

Paraformaldehyde (PFA) fixation was used to stain the nucleus and cytoskeleton. Briefly, PCUU nonwovens seeded with cells were fixed for 20 min in 4% PFA (Thermo Scientific) after washing three times with 1× PBS, followed by being permeabilized with 0.1% Triton X-100 for 30 min. Later, after washing three times with 1× PBS, cells on nonwoven materials were stained with DAPI, cat. #62247 Thermo Fisher Scientific and phalloidin (Phalloidin-iFluor 555 Reagent, cat #ab176756, Abcam, UK) by adding 400 cm^3^ of the staining solution (DAPI (1:1000) and phalloidin (1:1000)) to each well and incubated for 3 h at room temperature. Finally, nonwovens were mounted onto the glass slide and covered with a coverslip to visualize cells under a fluorescent microscope (Olympus IX83; Olympus, Tokyo, Japan).

Statistical analysis was performed with one-way analysis of variance algorithm. Summarized results from at least three independent in vitro biological runs are presented, and three replicates each were compared using post hoc Tukey’s tests.

##### 2.3.1.6 Film Degradation Assay

Three samples were investigated in the degradation assays: N_3_50, N_4_150, and N_5_150. Nonwovens (1 × 1 cm^2^) were incubated at 37 °C in a CO_2_ incubator in 10 cm^3^ 1× PBS buffer (pH 7,4) with gentle mixing for 14 days. For each material type, three samples were investigated for every degradation period. To analyze the effect of PBS, nonwovens were characterized for the change in their microstructure using SEM on days 0, 7, and 14. For SEM analysis, nonwovens were first mounted onto the stub and coated with platinum using a sputter coater for 45 s at 40 mA, as nonwovens were nonconductive. Further, the nonwovens were imaged using a Hitachi SU8010 scanning electron microscope at 5 kV with a 5 mm working distance for microstructural analysis. Images were analyzed using ImageJ 1.53t for nonwovens’ change in fiber diameter. ATR-FTIR spectra were recorded on a Thermo Scientific Nicolet iS5 FTIR spectrometer.

The errors of data, if not mentioned differently, were estimated as a standard deviation of a set of data.

## 3 Results and Discussion

This study was designed to investigate PCUU for electrospinning and evaluate the resulting nonwovens. We aimed to assess the effect of the polymer concentration and collector type on the nonwoven properties and biological assessment of the selected materials.

A detailed analysis of the starting material, casted PCUU, has been reported in our previous work.^62^ It allowed us to determine the polymer composition and enabled the selection of soluble structures of PCUU. Briefly, the obtained polymer was based on oligocarbonate diol (OCD) with an average molar mass (*M*_n_) of 3000 g·mol^–1^, estimated based on ^1^H NMR and confirmed by MALDI-TOF analysis. The molar excess of IPDI to the level of the OCD was equal to 3, which led to 18 wt % hard segments in the structure. Casted PCUU exhibited highly elastic properties with a tensile strength (σ) of 24 ± 3 MPa, an elongation at break (ε) of 990 ± 35%, and Young’s modulus (*E*) of 91 ± 7 MPa.

### 3.1 Preparation of Nonwovens: ES

The present study applied the ES process to PCUU solutions (3, 4, and 5 wt %) utilizing plate and drum collectors using 50 and 150 rpm. We determined the η range based on the experiments, which allowed us to perform the ES process (Table 1). In the case of viscosity lower than 20 mPa·s and higher than 100 mPa·s, we could not produce fibers regardless of other parameters applied in the process. Interestingly, a rapid rise in ν values from ∼99 to ∼282 mm^2^ s^–1^ for 5 and 6 wt % PCUU, respectively, was observed. These results were consistent with our observation that the PCUU at 6 wt % concentration was too viscous for the ES process to be effective. If the solution was too diluted, then the polymer fiber broke up into droplets before reaching the collector due to the effects of surface tension and insufficient overlapping of polymer molecules to the critical polymer concentration.^64^ However, if the concentration of the solution was too high, then fibers could not be formed due to the high viscosity, which made it difficult to control the solution flow rate through the capillary.^65^

**Table 1:** Kinematic Viscosity (ν), Density, and Dynamic Viscosity (η) of PCUU Solutions in DMF/THF: Influence of the Polymer Concentration in ES Solution

concentration of PCUU (wt %)	ν (mm^2^·s^–1^)	density (g·cm^–3^)	η (mPa·s)
2	12.00	0.91	10.96
3	25.11	0.92	23.10
4	49.90	0.92	45.67
5	99.39	0.92	91.34
6	282.40	0.93	261.16

### 3.2 Morphology of PCUU Nonwovens

**Figure 1:**
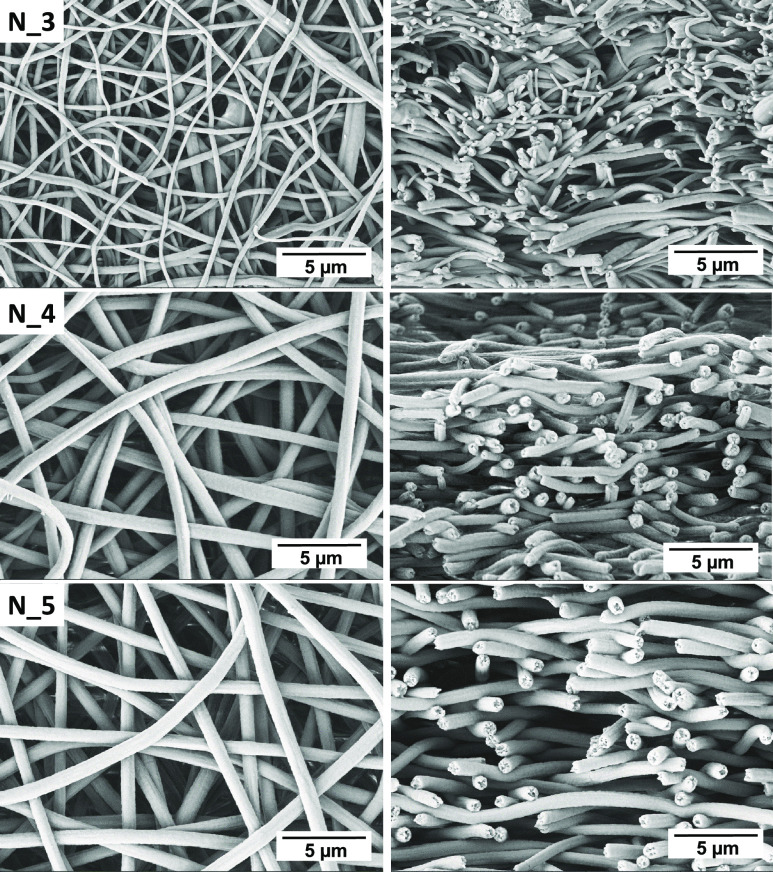
SEM micrographs showing the top (left) and cross-section (right) views of PCUU-based nonwovens obtained on a plate collector.

**Figure 2:**
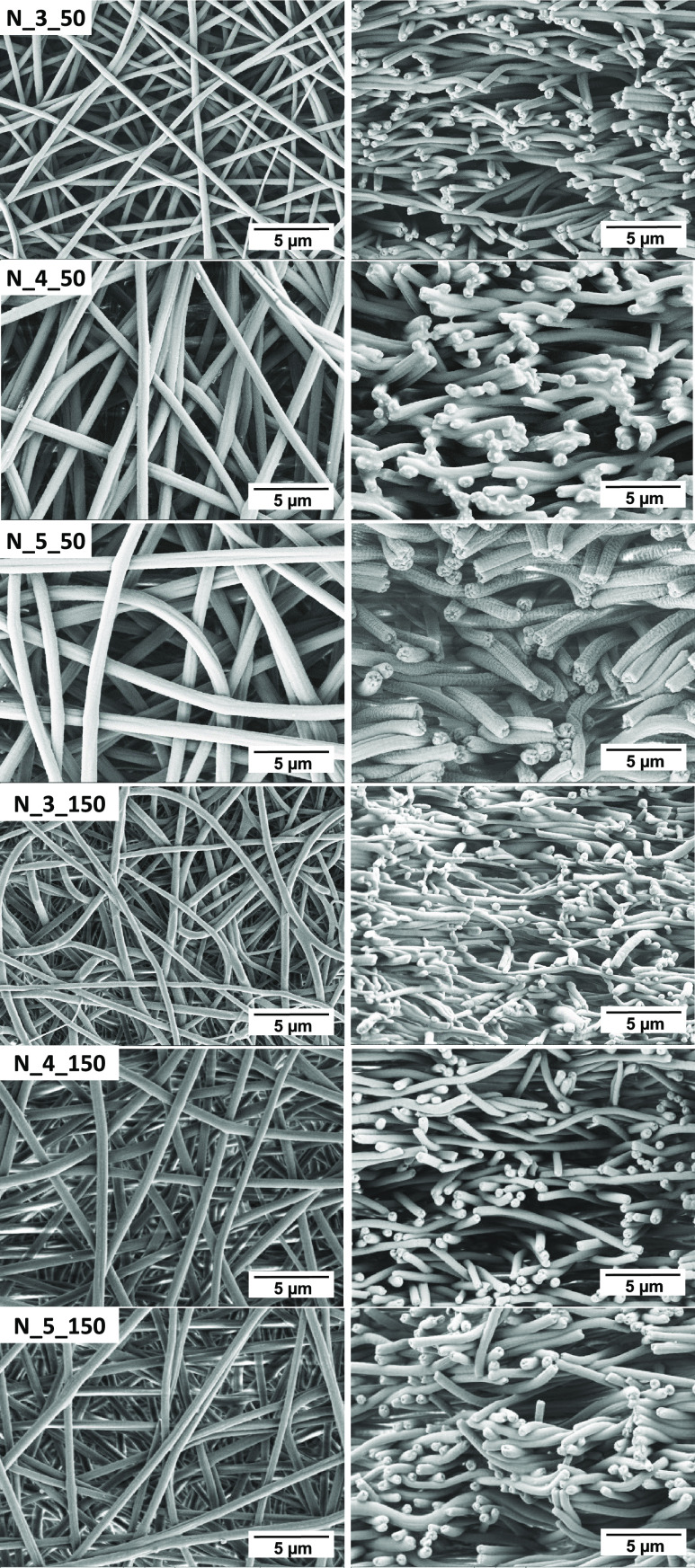
SEM micrographs showing the top (left) and cross-section (right) views of PCUU-based nonwovens obtained on a drum collector.

**Figure 3:**
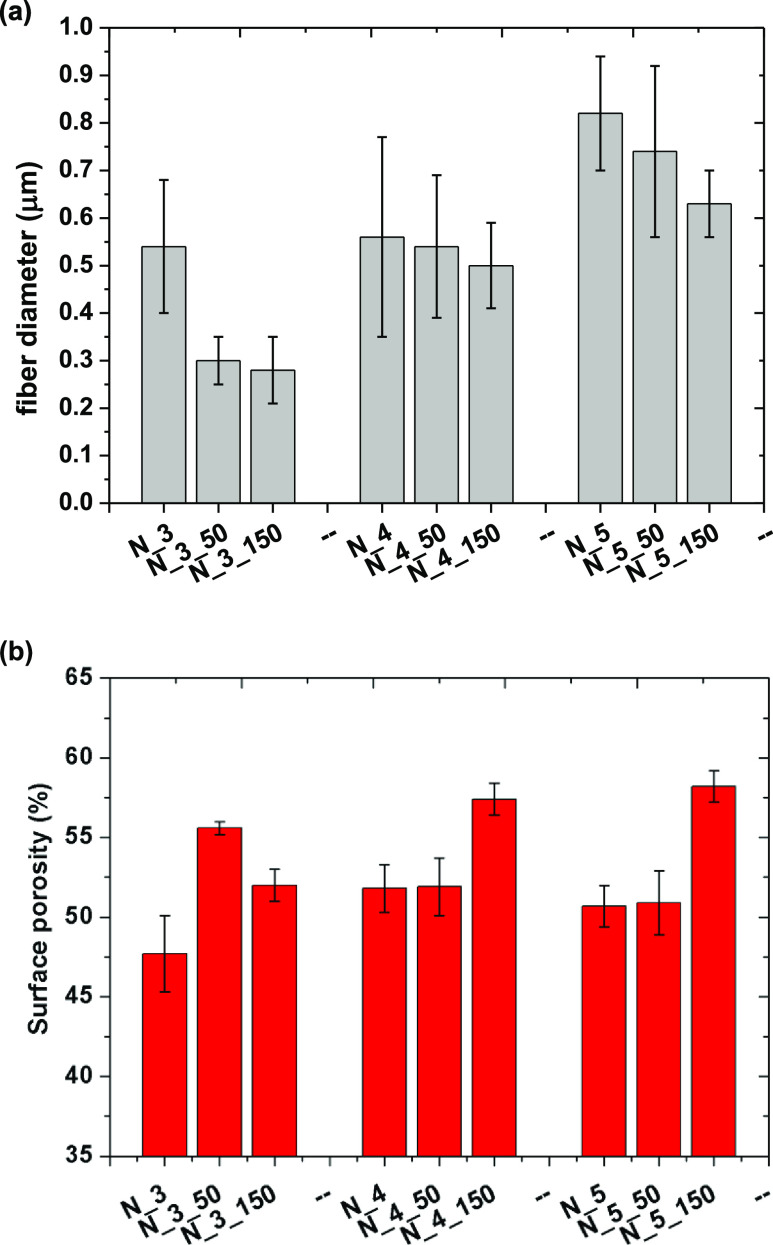
Fiber diameter (a) and surface porosity (b) of PCUU nonwovens calculated based on SEM images.

SEM images of all samples are presented in Figure 1 (plate collector) and Figure 2 (drum collector). The speed of the rotation influenced the diameter of the fibers. It has been shown that introducing the rotary motion of the drum leads to fibers with smaller diameters than in the case of usage of plate collectors,^66,67^ which is related to the stretching of the fiber during the ES process. The same behavior was observed in this study (Figure 3a). For example, the fiber diameter of PCUU nonwovens based on 3 wt % solutions obtained on the plate collector was 0.54 ± 0.14 μm, which decreased to 0.30 ± 0.05 and 0.28 ± 0.07 μm in the cases of 50 and 150 rpm, respectively. The same trends of fiber diameter of PCUUs were observed in the cases of usage of 4 and 5 wt % solutions—the diameter of fibers decreased with the rotation speed. Furthermore, the diameter of the fibers increased with an increase of the solution concentration, which was previously described for different polymer types in fiber formation processes.^65−68^ For example, Megelski et al. found that increasing the polystyrene concentration in THF increased the fiber diameter and narrowed the pore sizes.^69^ Furthermore, no significant change in the level of alignment of the fibers was observed between the various collector speeds, which is expected and related to the range of rotating speeds.^70−72^ The surface porosity of the nonwovens (Figure 3b) increased with an increase of the rotation speed, which was most probably assigned to the decrease of the diameter of the fibers and, in most of the cases, was the most pronounced for an applied speed of 150 rpm. Furthermore, no significant change in surface porosity as a function of polymer solution concentration was observed.

### 3.3 Thermal and Mechanical Properties of PCUU Nonwovens

**Figure 4:**
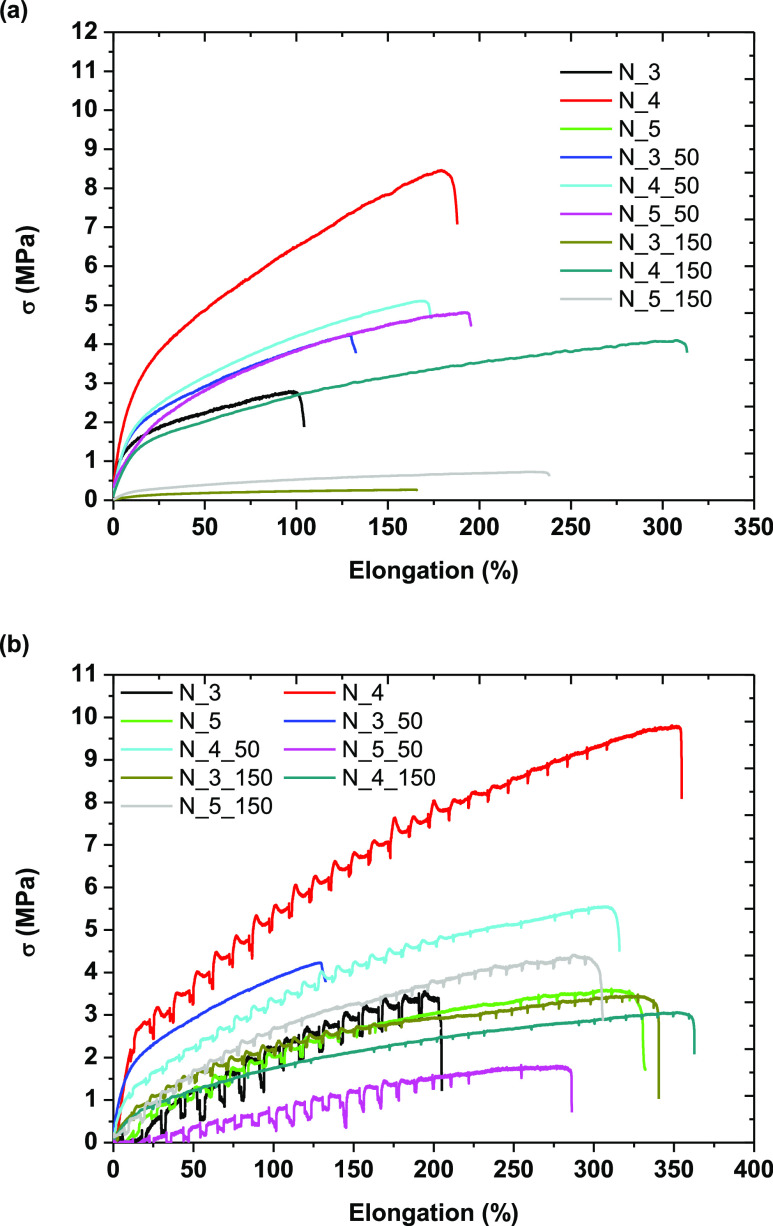
Strain–stress curves of nonwovens at (a) RT and (b) 37 °C.

The influence of the polymer concentration and collector type on the mechanical properties of the obtained PCUU nonwovens was determined by a tensile test. We performed tests at two temperatures: at room temperature (RT) and at 37 °C (as body temperature). The obtained results of Young’s modulus (*E*), tensile strength (σ), and elongation at break (ε) are listed in Table 2 and shown in Figures 4a (tests at RT), 4b (tests at 37 °C), and Figure S9 in the Supporting Information.

**Table 2:** Mechanical Properties of PCUU-Based Nonwovens: Young’s Modulus (*E*), Tensile Strength (σ), and Elongation at Break (ε) at Room Temperature (RT) and 37 °C

temperature	sample name	*E* (MPa)	σ (MPa)	ε (%)
RT	N_3	8.1 ± 2.5	2.5 ± 0.5	87 ± 23
	N_4	27.6 ± 7.3	8.1 ± 1.8	177 ± 28
	N_5	25.3 ± 1.6	7.1 ± 0.7	202 ± 24
	N_3_50	9.3 ± 0.7	4.2 ± 0.2	133 ± 17
	N_4_50	18.5 ± 2.7	5.3 ± 0.6	179 ± 50
	N_5_50	10.0 ± 1.8	4.9 ± 0.5	206 ± 32
	N_3_150	2.2 ± 1.0	0.3 ± 0.1	193 ± 33
	N_4_150	11.8 ± 0.5	4.0 ± 0.3	288 ± 40
	N_5_150	5.7 ± 1.3	0.7 ± 0.0	232 ± 13
37 °C	N_3	5.3 ± 1.5	3.5 ± 1.1	216 ± 67
	N_4	25.5 ± 6.2	9.6 ± 1.2	374 ± 53
	N_5	6.6 ± 2.2	3.7 ± 0.9	292 ± 50
	N_3_50	5.2 ± 2.3	3.0 ± 0.1	283 ± 65
	N_4_50	8.2 ± 1.9	3.8 ± 1.6	189 ± 110
	N_5_50	1.2 ± 0.1	1.0 ± 0.3	328 ± 52
	N_3_150	5.5 ± 2.3	3.4 ± 0.3	316 ± 54
	N_4_150	5.4 ± 1.1	2.5 ± 0.5	262 ± 75
	N_5_150	4.5 ± 2.0	4.7 ± 0.4	287 ± 40

Data obtained based on mechanical testing are not fully consistent, but several trends could be observed. At room temperature, *E* was 5.7–26.7 MPa, σ was 0.3–9.6 MPa, and ε was 90–290% MPa. With an increase in the rotation speed of the collector, we observed a decrease in *E* and σ, whereas the elongation at break increased. The highest values of mechanical parameters were observed in the case of 4 wt % solutions compared to samples based on other concentrations. Theoretically, the orientation within fibers on the molecular level increases with decreasing fiber diameter, resulting in a higher mechanical strength.^73,74^ Therefore, it is expected that with decreasing fiber diameter, the tensile strength of the electrospun fiber increases.^75^ However, some reports claimed that the nanofibers’ tensile strength increased with the fiber diameter increase.^76−78^ This behavior was observed for a polyurethane-based electrospun fiber scaffold, which has a chemical nature similar to our PCUUs. Han et al. concluded that the lower tensile strength in the case of small-diameter fibers was caused by weaker junctions of interconnected fibers.^76^ However, it could also have resulted from defects in individual fibers. Furthermore, inconsistent results could have also been caused by any interactions between fibers (slipping of fibers one over another, number of bonding points, cross-linking), variation in the fiber diameter, and fiber orientation within the nonwoven matrix.^79^

**Figure 5:**
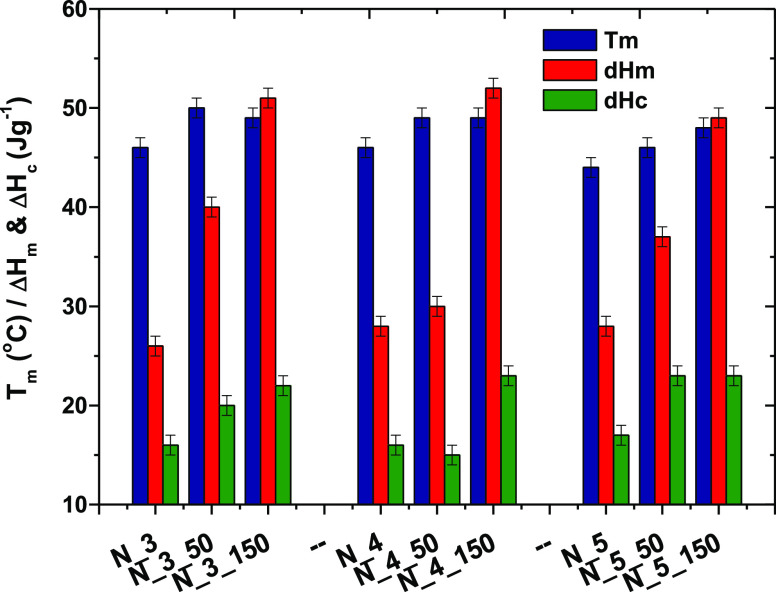
Thermal properties of PCUU nonwovens −*T*_m_, Δ*H*_m_, and Δ*H*_c_.

**Figure 6:**
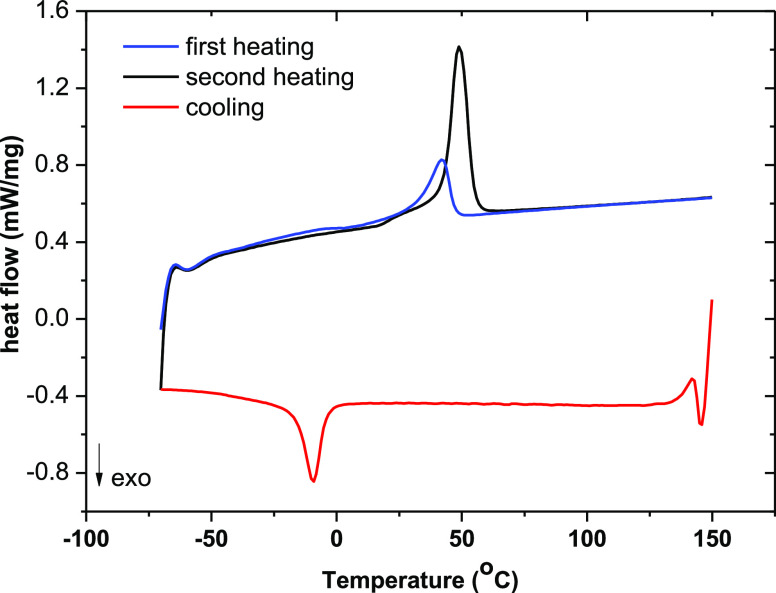
Exemplary DSC curves for N_4_150.

To determine the influence of the polymer concentration and the collector type on the thermal properties of the obtained PCUU nonwovens, DSC and DMTA tests were performed. Results based on DSC are presented in Figure 5 and Table S3 in the Supporting Information. Exemplary DSC curves showing two cycles of heating and cooling are shown in Figure 6.

It was found that the electrospun samples of PCUUs compared to casted samples of the same material reported in^62^ had higher *T*_g_ values, which suggested a reduction in the separation of microphases in the obtained fibers.^80^ This phenomenon was previously observed in the cases of electrospun PU^81^ and polylactide nanofibers.^82^ In the case of PCUUs, which contain soft (carbonate) and hard (urethane and urea) segments, the hard segment physically cross-links the soft segment and therefore increases the *T*_g_ of the material. This phenomenon is limited by microphase separation; thus, a higher *T*_g_ of the soft segment means less microphase separation. In fact, the fast evaporation of solvent and immediate solidification of PCUU during the ES process limited the microphase separation of soft and hard segments. We also observed a decrease of *T*_g_ when a rotary collector was applied. The strain-induced crystallization of PCUU during the ES process is higher at higher rotational rates. The strain-induced crystallization can result in partial microphase separation, and thus, the *T*_g_ of the soft segment shifted down. Values of Δ*H*_m_ of nonwovens in the first run (18–40 J·g^–1^) were higher than in casted materials (18 J·g^–1^),^62^ which indicates an increase in the order of the structure and, consequently, an increase in the crystalline phase. This is a consequence of strain-induced crystallization, which occurs during ES.^83^ Furthermore, *T*_m_, Δ*H*_m_ in the first run, and Δ*H*_c_ for the nonwovens obtained on the plate collector had lower values compared to the materials obtained on the drum collector. It indicates an increase of the order of the polymer thread deposited on the rotary collector, thus increasing the content of the crystalline phase. This increase is related to strain-induced crystallization, which occurs when stretching forces are applied to the polymer stream.

Thermal properties were also investigated via DMTA measurements (Figure S10 in the Supporting Information), and *T*_g_ and *T*_m_ were determined. As presented in Table 3, *T*_g_ (determined by tan delta) decreased from −13 ± 1 °C for N_3 to −18 ± 1 °C for N_5_150. Furthermore, a clear influence of the PCCU concentration on the *T*_g_ concentration could not be detected. When *T*_g_ was determined by the storage modulus *E*′, *T*_g_ ranged between −16 and −18 °C, without a significant influence of preparation parameters or concentration on thermal properties. The melting transition of PCUU samples was slightly influenced by the changed collector speed (*T*_m_ increased from 48 ± 1 °C for N_3_50 to 51 ± 1 °C for N_3_150), whereas the PCUU concentration did not affect the resulting *T*_m_.

**Table 3:** *T*_g_ and *T*_m_ of PCUUs Based on DMTA^a^

	*T*_g_	*T*_g_	*T*_m_
	tan δ	*E*′	*E*′
sample	°C	°C	°C
N_3	–13 ± 1	–16 ± 1	n.d.
N_4	–14 ± 1	–17 ± 1	n.d.
N_5	–14 ± 1	–17 ± 1	n.d.
N_3_50	–14 ± 1	–15 ± 1	48 ± 1
N_4_50	–16 ± 1	–18 ± 1	49 ± 1
N_5_50	–17 ± 1	–18 ± 1	n.d.
N_3_150	–17 ± 1	–17 ± 1	51 ± 1
N_4_150	–18 ± 1	–18 ± 1	50 ± 1
N_5_150	–18 ± 1	–18 ± 1	50 ± 1

a n.d., no data. *T*_m_ could not be detected as the sample broke during heating.

**Figure 7:**
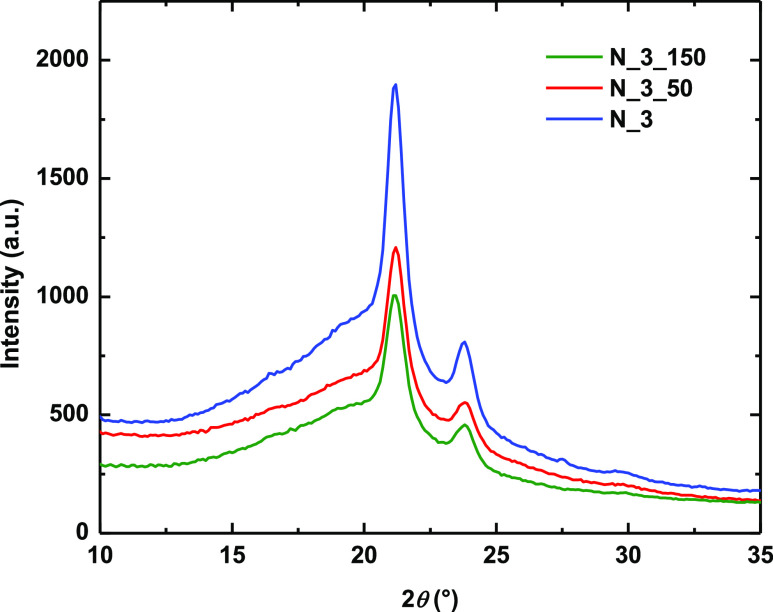
WAXS scattering plots shown are exemplary for nonwovens N_3, N_3_50, and N_3_150.

WAXS analysis (Table 4 and Figure 7) was used to determine the effect of ES collector speed on the crystallization of PCUU fibers as indicated by the DSC analysis. Surprisingly, there were no significant differences between the degree of crystallinity (DOC) and crystal size (*l*_c_) of the tested materials. Furthermore, no trends in the relation of DOC or *l*_c_ with collector type or concentration of the solution were observed (Table 4). The same problem was faced by Kołbuk et al., who did not observe induced crystallization upon collecting fibers on a rotating drum when the rotating speed was 1000 rpm.^84^ Other authors found that the induction of fiber alignment occurs with rotation speeds of over 1500 rpm,^85^ which is a much faster speed than we used in our investigations. Therefore, it is possible that we did not observe both the alignment of fibers and the increase in the crystallinity of fibers in the tested systems with the proposed process parameters.

**Table 4:** Average Degree of Crystallinity DOC (%) and Crystal Size *l*_c_ (nm) Determined Based on WAXS

collector type	sample name	average DOC (%)	average *l*_c_ (nm)
plate collector	N_3	28.8 ± 2.0	13.2 ± 0.4
	N_4	27.7 ± 0.6	11.5 ± 0.1
	N_5	28.0 ± 1.1	12.5 ± 0.1
rotating drum collector	N_3_50	28.6 ± 0.4	12.4 ± 0.1
	N_4_50	30.9 ± 0.6	12.5 ± 0.2
	N_5_50	25.0 ± 0.1	11.1 ± 0.0
	N_3_150	27.1 ± 0.3	12.4 ± 0.1
	N_4_150	28.4 ± 0.8	9.1 ± 0.2
	N_5_150	29.6 ± 4.0	11.6 ± 0.1

### 3.4 Shape-Memory Effect Investigation

Based on promising ES applications of shape-memory polymers, we decided to conduct SME studies of our materials. The motivation for this investigation was based on the fact that the casted PCUUs exhibit very good shape-memory properties, with a shape-recovery ratio (*R*_r_) of 99.6 ± 0.1% and a shape-fixity ratio (*R*_f_) of 95.8 ± 0.1%.^62^ In the present studies, similar shape-memory parameters were observed for the nonwovens obtained by ES. In the case of samples prepared on a plate collector, we observed good shape-memory performance with *R*_r_ around 99% and *R*_f_ around 96% (Table 5). Diagrams of one-way shape-memory performance of N_3 and N_4_150 are shown in the Supporting Information in Figures S11 and S12, respectively. Samples were elongated until they reached 0.5 N (which was approximately 200% of the relative elongation). Then, the stress was held for 10 min, followed by cooling the sample to −5 °C, while the temporary shape was fixed. Afterward, samples recovered to their original shape by heating to 50 °C. Interestingly, nonwovens collected on a drum collector were characterized by high *R*_f_ but very low *R*_r_ and also expansion while heating (Figure S12, Supporting Information). This is probably related to the fibers melting, which causes shrinkage and rearrangement of the nonwovens at 58 °C. In those samples, preheating at 8 °C in the programming step was probably the recovery step of the ES-induced shape-memory effect. During ES, the fibers underwent deformation, followed by a contraction due to the decreased molecular mobility caused by the rapid solvent evaporation; thus, the ES process was identified to be like the programming cycle, while the contraction effect was identified as the recovery phase.^86,87^ However, more investigations related to the shape-memory effect of PCUU samples obtained on a drum collector need to be done, with a detailed study of temperatures of programming and recovery.

**Table 5:** Shape-Memory Effect Results: Shape-Recovery Ratio (*R*_r_) and Shape-Fixity Ratio (*R*_f_)

sample name	*R*_r_ (%)	*R*_f_ (%)
N_3	98.8 ± 0.5	96.3 ± 0.5
N_4	99.3 ± 0.5	95.7 ± 0.1
N_5	99.1 ± 0.5	95.8 ± 0.2

### 3.5 Incubation of Fibers in PBS Buffer

**Figure 8:**
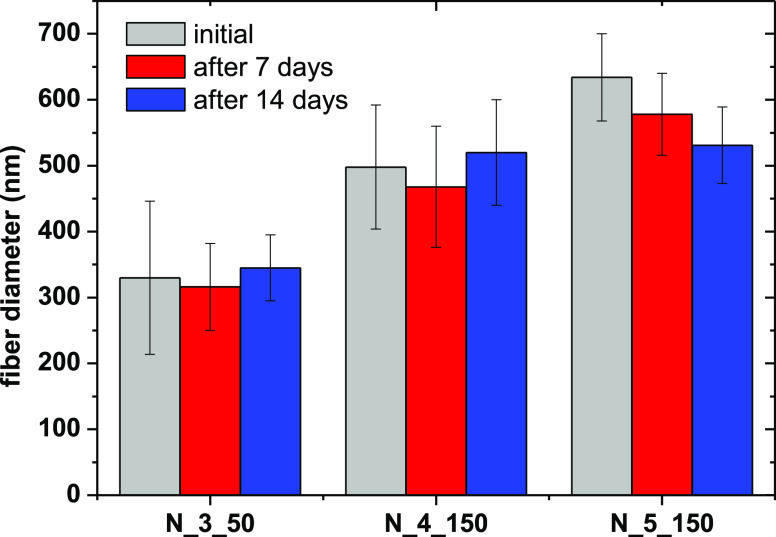
Fiber diameter (nm) of fibers before and after incubation in BPS buffer for 7 and 14 days.

Incubation of three selected PCUU nonwovens with 1× PBS buffer for 14 days was performed to study the stability of the nonwovens under physiological conditions. Only a slight change in the fiber diameter was observed on the seventh and 14th days, demonstrating the stability of PCUU nonwovens in PBS (Figure 8 and Table S4 in the Supporting Information). PCUU fibers were evaluated by morphological changes assessed by SEM (Figure S13 in the Supporting Information) and changes in the chemical bonds and composition measured by FTIR (Figures S14–S16 in the Supporting Information). As in the case of the casted films obtained from PCUUs,^88^ the nonwoven materials based on the same polymer showed good resistance against hydrolysis in a period of 14 days.

### 3.6 Biological Evaluations

**Figure 9:**
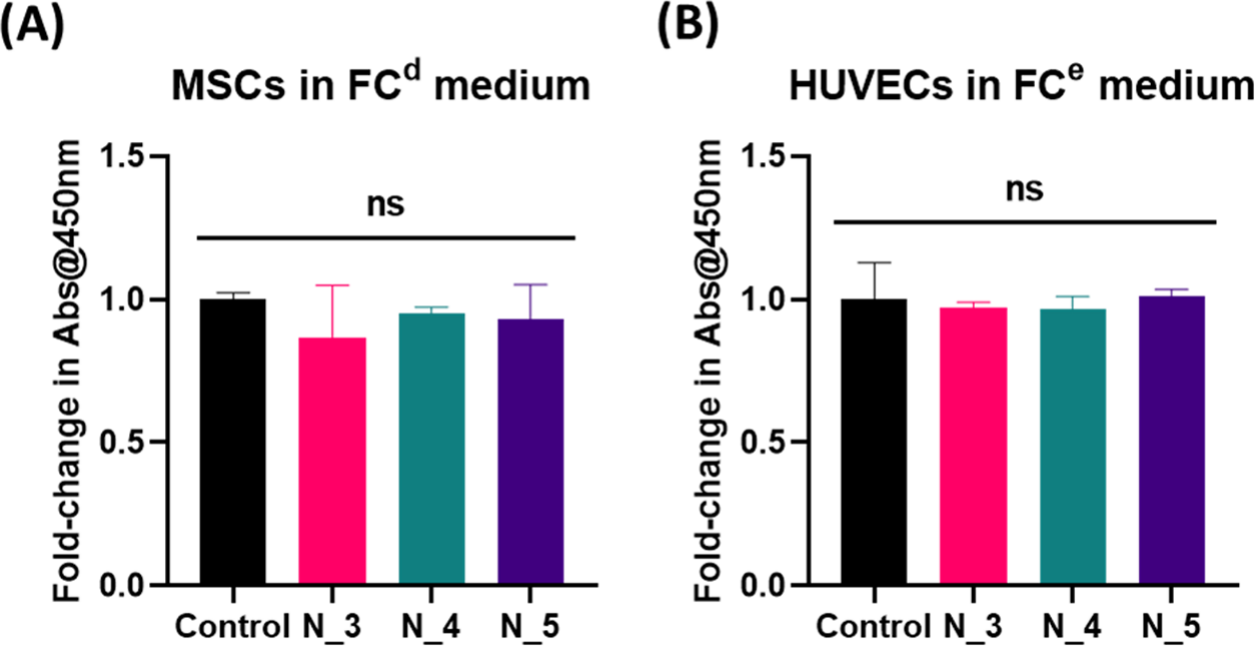
CCK-8 assay for MSCs (A) and HUVECs (B) grown in FC-DMEM (FC^d^) and FC-EGM-II (FC^e^) mediums for 72 h, respectively (indirect incubation). No significant changes were observed (*p* > 0.05).

Electrospun fibers have been previously successfully used in many tissue engineering applications, for example, in neural,^89^ tendon,^90^ bone,^91^ and vascular applications.^92^ Interestingly, due to the ability to prevent smooth muscle cell migration, as well as to form aligned scaffolds with anisotropic mechanical and biological properties, the ES nonwovens received high interest in the field of vascular grafts. In order to prevent thrombolysis, there is a need to precondition the materials with endothelial cells before implantation. Previously, the application of ES nonwovens demonstrated a more favorable cell attachment associated with their high availability of structure.^93,94^ To study the cytocompatibility of the PCUU nonwovens, a nonwoven conditioned (FC) medium was obtained by incubating nonwovens with EGM-2 and DMEM medium for 24 h, upon which cell proliferation assays with HUVECs and MSCs were performed. Incubation of nonwovens with the medium allowed for complete saturation of nonwovens with the medium and the permeation of loosely bound solvable components inside the medium’s fibers. Cell proliferation of MSCs and HUVECs grown in FC^e^ and FC^d^ mediums demonstrated no significant differences in terms of cell numbers as compared to those grown in unconditioned EGM-2 and DMEM (controls) (Figure 9A,9B, respectively). This validates the inertness, stability, and lack of leakage of cytotoxic components from PCUU nonwovens obtained on the plate collector.

**Figure 10:**
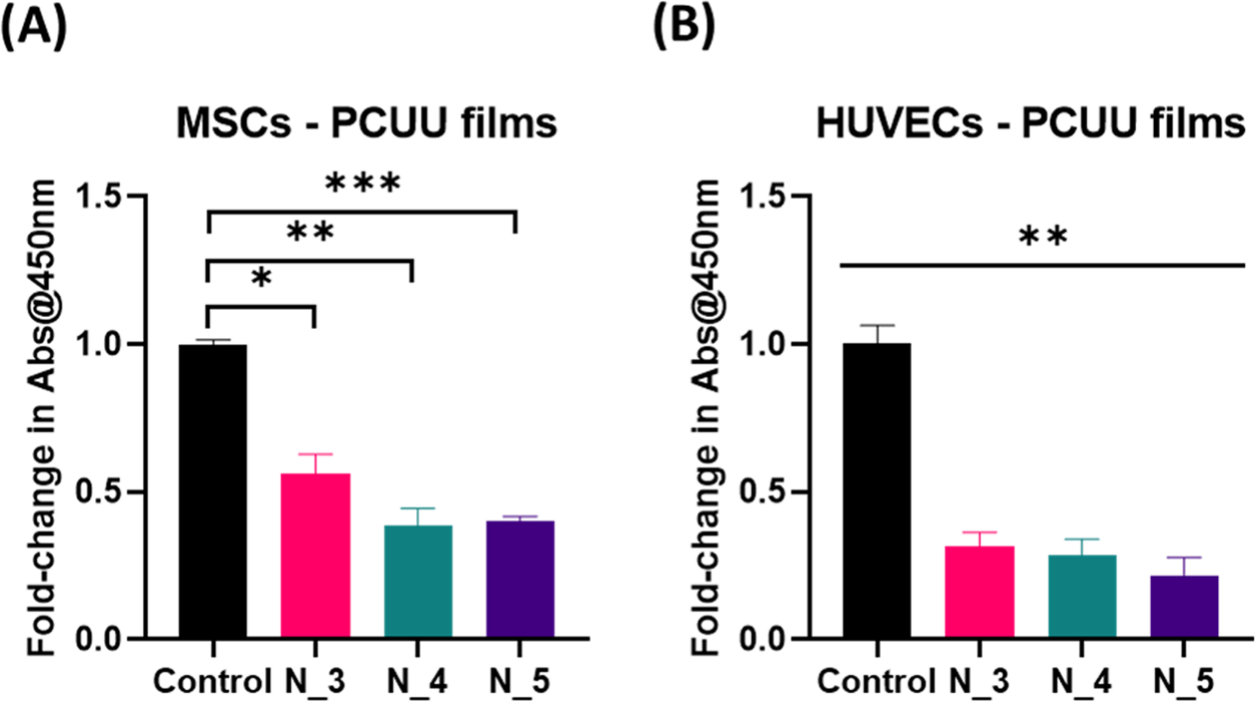
CCK-8 assay for MSCs (A) and HUVECs (B) directly seeded on PCUU nonwovens and cultured for 2 days (* indicates a *p*-value < 0.05; ** indicates a *p*-value *p* < 0.01, *** indicates a *p*-value < 0.001).

The ability of the obtained PCUU nonwovens to support cell attachment and proliferation was studied by seeding cells (MSCs and HUVECs) directly onto the nonwovens. Cell proliferation for both of the MSCs and HUVECs seeded onto the PCUU nonwovens on the plate collector was significantly lower than the control samples (tissue culture polystyrene, TCP) as shown in Figure 10A,10B, respectively. As TCP is a standard and optimal substrate for cell attachment and cell growth, lower proliferation rates are likely explained by fewer cell binding sites available on PCUU nonwoven surfaces.

**Figure 11:**
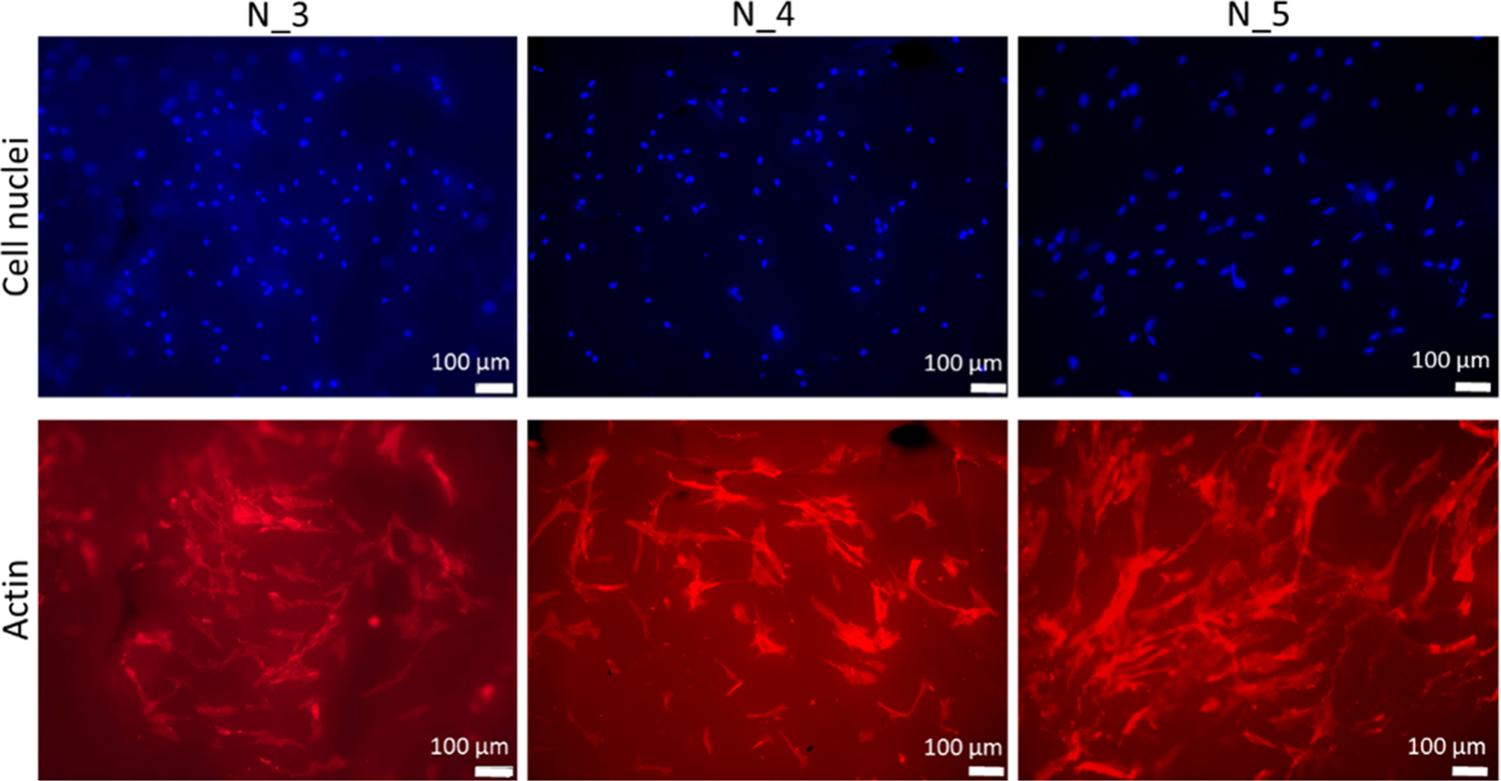
Phalloidin (staining of actin cytoskeleton) and DAPI (staining cell nuclei) staining of MSCs cultured on PCUU nonwovens for 2 days.

**Figure 12:**
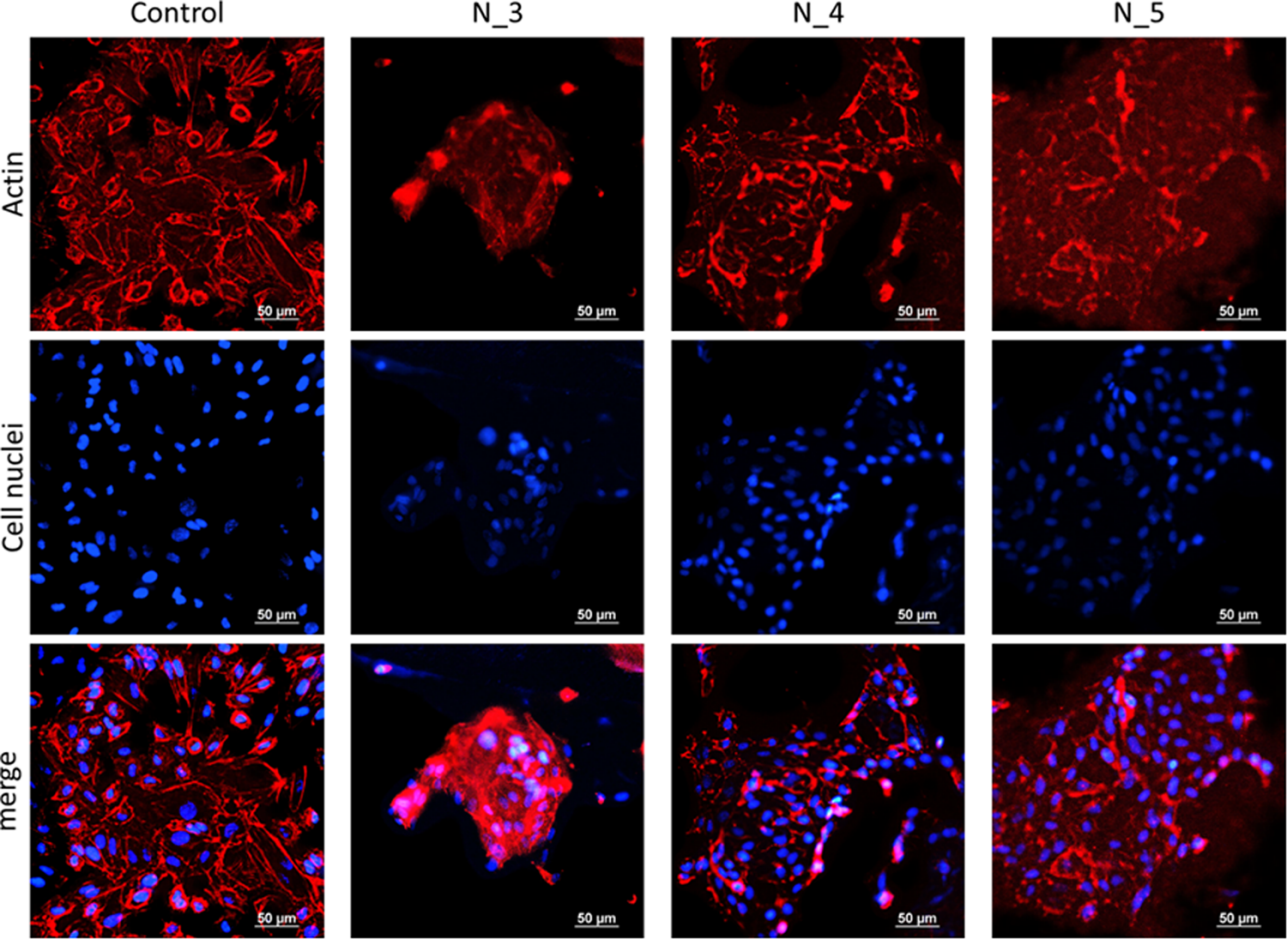
Phalloidin (staining of actin cytoskeleton) and DAPI (staining cell nuclei) staining of HUVECs cultured on PCUU nonwovens for 2 days. Tissue culture polystyrene (TCP) was used as a control.

Furthermore, MSCs (Figure 11) and HUVECs (Figure 12) cultured on PCUU nonwovens were visualized by fluorescence microscopy upon staining for their actin cytoskeleton (by phalloidin staining) and cell nuclei (by DAPI staining). MSCs attached and spread on PCUU materials, exhibiting a spindle shape, as expected for well-adhered MSCs (Figure 11). HUVECs also attached to PCUU nonwovens; however, they aggregated and exhibited a more diffusive staining of their cytoskeleton (Figure 12). This suggests that HUVECs attached less well on PCUU fibers as compared to TCP.

**Figure 13:**
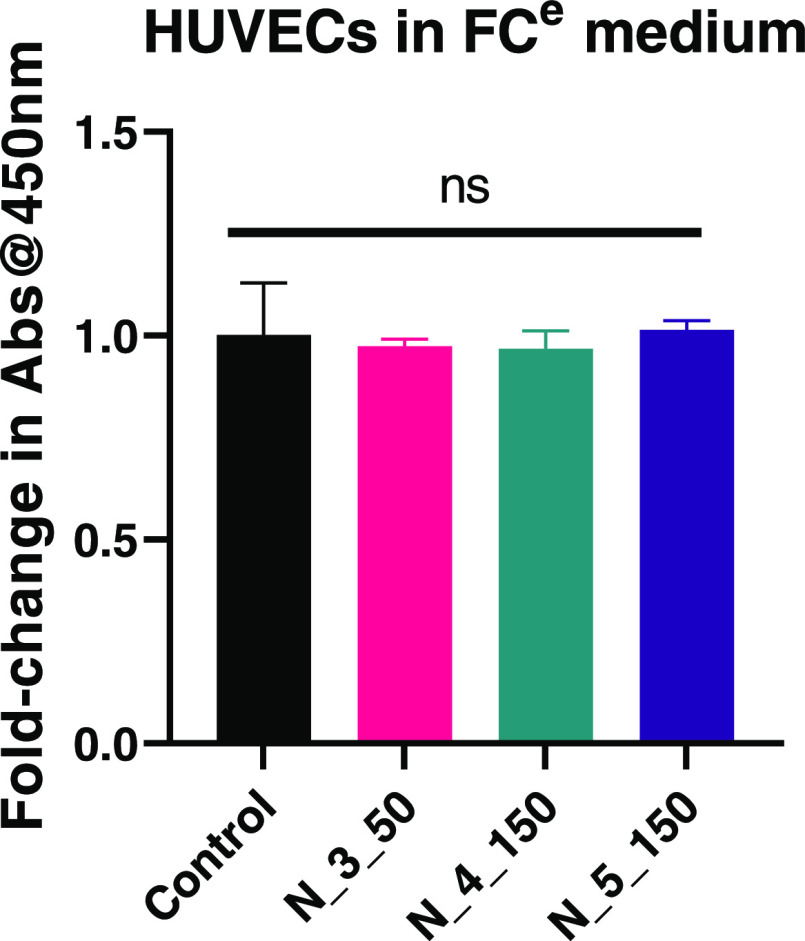
CCK-8 assay of HUVECs cultured for 72 h in the FC-EGM-2 (FC^e^) medium conditioned by PCUU nonwovens obtained with a rotary collector. No significant changes were observed (*p* > 0.05).

**Figure 14:**
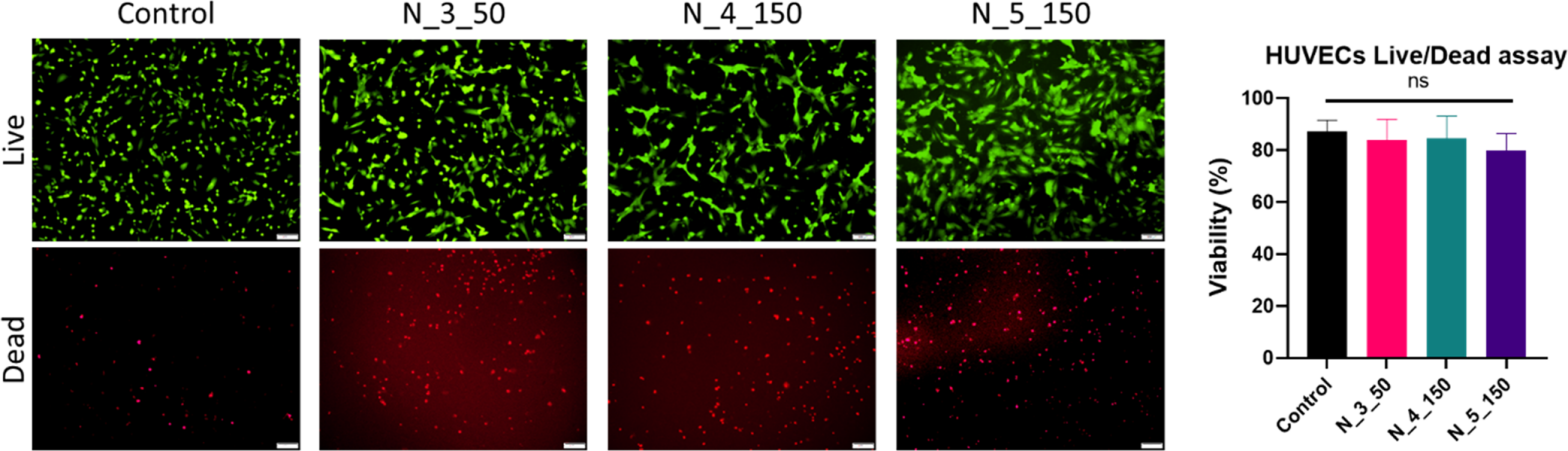
Live (green)/dead (red) cell staining of HUVECs cultured in the presence of nonwovens obtained with a rotary collector.

Similarly, when indirect cytotoxicity assays were performed with HUVECs cultured in the medium conditioned by nonwovens obtained on the rotary collector (exemplary for PCUU_3_50, PCUU_4_150, and PCUU_5_150), good cytocompatibility was observed (Figure 13). However, when HUVECs or MSCs were directly seeded onto the nonwovens, no cell attachment was observed. Hence, cell visualization was performed. Nonetheless, HUVECs cultured in a culture medium submerged with the nonwovens obtained on the rotary collector showed no adverse responses, as evident from the comparable viability to the control medium derived from live/dead cell staining (Figure 14).

No attachment of the cells to the surface of rotary collector-based nonwovens could be assigned to the differences in the morphology of the surface of samples based on this type of collector. When we analyze the surface porosity (Figure 3b), we could clearly see that for the investigated samples N_3_50, N_4_150, and N_5_150 it was over 55%, whereas all samples based on plate collector did not reach that value.

As MSCs did not attach to the nonwovens, we did not perform any further cytotoxicity investigation with samples obtained with a rotary collector and these cells.

## 4 Conclusions

In this study, we aimed to assess the effect of the polymer concentration and collector type on the nonwoven properties and the biological assessment of the selected materials. PCUU nonwovens of fiber diameters in the range from 0.28 ± 0.07 to 0.82 ± 0.12 μm were obtained with an average surface porosity of around 50–60%. The obtained nonwovens were characterized by a broad range of tensile strengths (in the range of 0.3–9.6 MPa), elongation at break (90–290%), and Young’s modulus (5.7–26.7 MPa), which strongly depended on the collector type and solution concentration. Thermal characterization showed that during ES the degree of crystallinity increases most probably due to strain-induced crystallization. Furthermore, samples collected on the plate collector were able to show a shape-memory effect with a shape-recovery ratio *R*_r_ of around 99% and a shape-fixity ratio *R*_f_ of around 96%. Biological evaluation validated the inertness, stability, and lack of cytotoxicity of PCUU nonwovens. Moreover, two types of cells, MSCs and HUVECs, were seeded on the materials, which revealed that they are able to grow on the surface of the nonwovens collected on the plate collector. Interestingly, both types of cells did not attach to the surface of nonwovens obtained with the rotary collector. This could be assigned to the differences in the morphology, specifically in the surface porosity, which was over 55% in the case of nonwovens obtained by the rotary collector and used for the biological study. Further investigations with a rotary collector will be performed to find the conditions of ES, which would lead to optimal surface porosity. Based on the advantages of the morphology, as well as tensile and shape-memory properties, the nonwovens obtained by the plate collector are potentially expected to be used in the high-performance membranes for cardiovascular purposes.

## Supporting Information Available

Scheme of the synthesis of PCUU; amounts of reagents used in the syntheses of the PCUU film; ^1^H NMR spectra of all products; FTIR spectra of all products; values of porosity and thickness of the obtained electrospun PCUU nonwovens and average fiber diameter; diagram of the mechanical properties of samples obtained from solutions with various concentrations and electrospun with various speeds of the collector at RT and 37 °C; table containing thermal properties of PCUU nonwovens based on DSC curves; DMTA plots of the storage modulus *E*’ and tan delta as a function of temperature; diagram of one-way shape-memory performance of N_3; diagram of one-way shape-memory performance of N_4_150 with calculated *R*_r_ and *R*_f_ in each cycle of the measurement; fiber diameter based on SEM analysis of PCUU nonwovens incubated in PBS buffer for 0, 7, and 14 days; SEM analysis of PCUU nonwovens incubated in PBS buffer for 0, 7, and 14 days; and FTIR spectra of N_3_50 and N_5_150 samples before and after biodegradation treatment (for 7 and 14 days) (PDF)
