# Supplementary material for: Electrospun Poly(carbonate-urea-urethane)s Nonwovens with Shape-Memory Properties as a Potential Biomaterial
Source: ACS Biomater Sci Eng. 2023 Nov 30;9(12):6683–97. doi: 10.1021/acsbiomaterials.3c01214 (PMC10716822; doi:10.1021/acsbiomaterials.3c01214)
Supplement: Supplementary file 1 — ab3c01214_si_001.pdf [file ab3c01214_si_001.pdf]

Supporting Information

Electrospun poly(carbonate-urea-urethane)s nonwovens with shape-memory properties as a potential biomaterial

Karolina Rolińska[1,2], Hadi Bakhshi[3], Maria Balk[4], Anna Blocki[5,6,7], Amit Panwar[5,6,7], Michał Puchalski[8], Michał Wojasiński[9], Magdalena Mazurek-Budzyńska[1]*

[1] Faculty of Chemistry, Warsaw University of Technology, Noakowskiego 3, 00-664 Warsaw, Poland;

[2] Faculty of Chemistry, University of Warsaw, Pasteura 1, 02-093 Warsaw, Poland;

[3] Department of Life Science and Bioprocesses, Fraunhofer Institute for Applied Polymer Research IAP, Geiselbergstraße 69, 14476 Potsdam, Germany;

[4] Institute of Active Polymers, Helmholtz-Zentrum Hereon, Kantstraße 55, 14513 Teltow, Germany;

[5] Institute for Tissue Engineering and Regenerative Medicine, The Chinese University of Hong Kong, HKSAR;

[6] School of Biomedical Sciences, Faculty of Medicine, The Chinese University of Hong Kong, HKSAR;

[7] Center for Neuromusculoskeletal Restorative Medicine, The Chinese University of Hong Kong, HKSAR;

[8] Institute of Material Science of Textiles and Polymer Composites, Faculty of Material Technologies and Textile Design, Lodz University of Technology, ul. Żeromskiego 116, 90-924 Łódź, Poland;

[9] Faculty of Chemical and Process Engineering, Department of Biotechnology and Bioprocess Engineering, Laboratory of Biomedical Engineering, Warsaw University of Technology, Waryńskiego 1, 00-645 Warsaw, Poland.

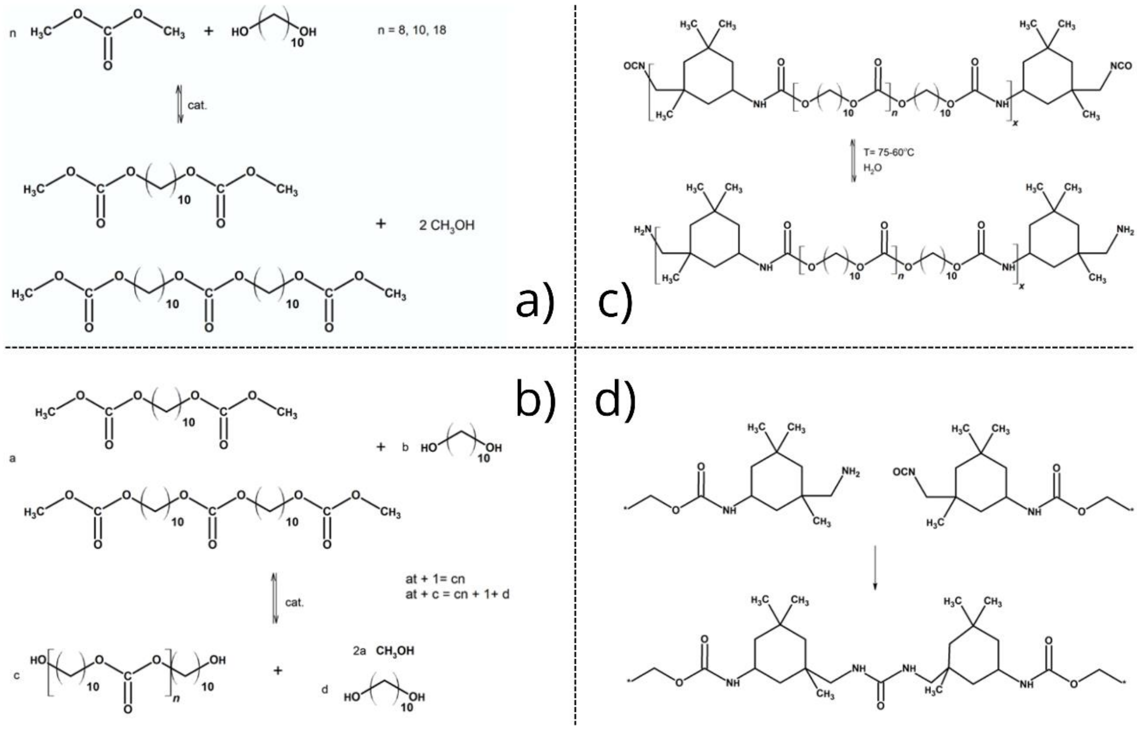

Scheme S1. Synthesis of PCUUs consisting of the following steps: a) alkylene bis(methyl carbonate)s (BMC); b) oligo(alkylene carbonate) diols (OSD); c) urethane prepolymers; and d) poly(carbonate-urethane-urea)s (PCUU).

NMR results for synthesized PCUU compounds:

BMC: ^{1}H NMR ($CDCl_3$. 400MHz): δ (ppm) = 4.12 (t. 4H, C(O)OCH_2). 3.77 (s. 6H. CH_3O). 1.63 (m. 4H. $OCH_2CH_2CH_2CH_2CH_2$). 1.33 (m. 4H. $OCH_2CH_2CH_2CH_2CH_2$). 1.26 (m. 8H. $OCH_2CH_2CH_2CH_2CH_2$).

FTIR (ATR): 2920, 2850, 1750, 1440, 1280, 1260, 950, 930, 790, 710 cm^{-1}.

OCD: ^{1}H NMR: ($CDCl_3$, 400MHz): δ (ppm)= 4.10 (t. 4H. C(O)OCH_2), 3.62 (t. 4H. CH_2OH). 1.65 (m. 4H. $HOCH_2CH_2CH_2CH_2CH_2$), 1.55 (m. 4H. $HOCH_2CH_2CH_2CH_2CH_2$), 1.53 (m. 4H. $OCH_2CH_2CH_2CH_2CH_2$), 1.32 (m. 4H. $OCH_2CH_2CH_2CH_2CH_2$), 1.27 (m. 4H. $OCH_2CH_2CH_2CH_2CH_2$) .

FT-IR (ATR): 3450, 2920, 2850, 1740, 1470, 1400, 1340, 1280, 1250, 1030, 940, 790 cm^{-1}.

Urethane prepolymer: FT-IR (KBr): 3670, 3380, 2960, 2910, 2260, 1750, 1720, 1240, 790 cm^{-1}.

PCUU: FT-IR (ATR): 3670, 3380, 2960, 2910, 1740, 1720, 1240, 790, 720, 430 cm^{-1}.

Table S1. Amount of reagents used in the syntheses of the PCUU film.

Synthesis step	The name of the substrate	Mass; g
BCM	DMC	1000
	1,10-decanediol	250
OCD	BCM	150
	1,10-decanediol	56.5
Urethane Prepolymers	OCD	15.3
	IPDI	3.4

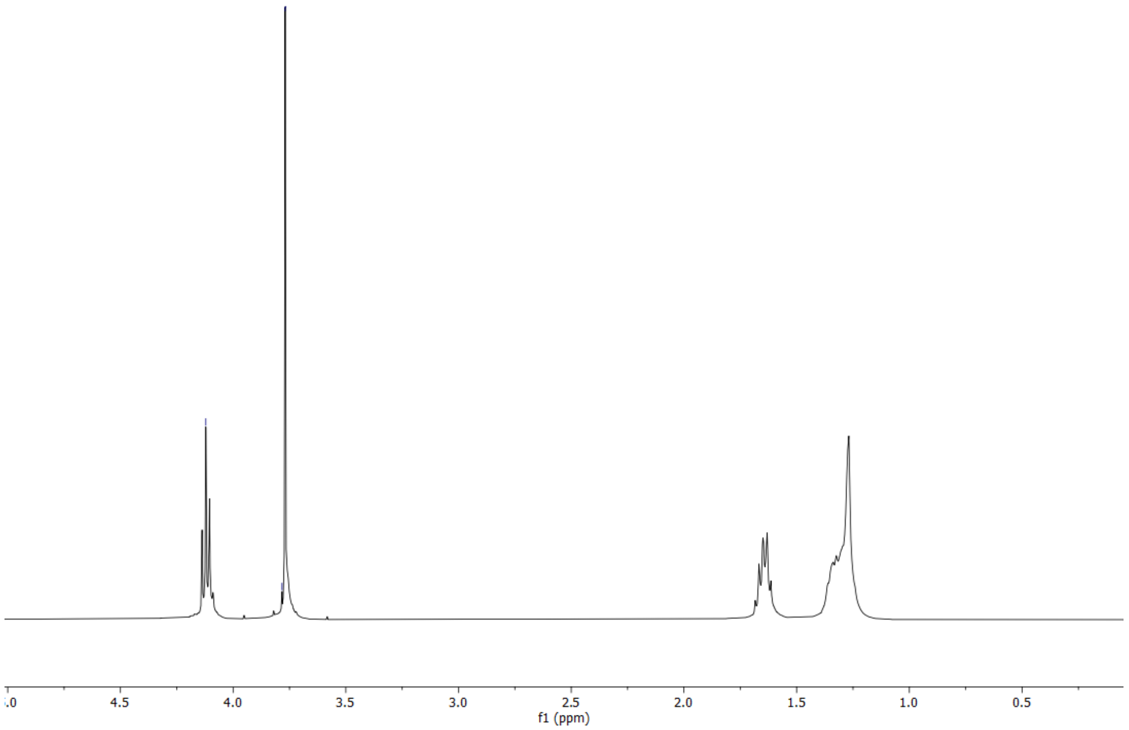

Figure S1. ^{1}H NMR spectrum of the BMC.

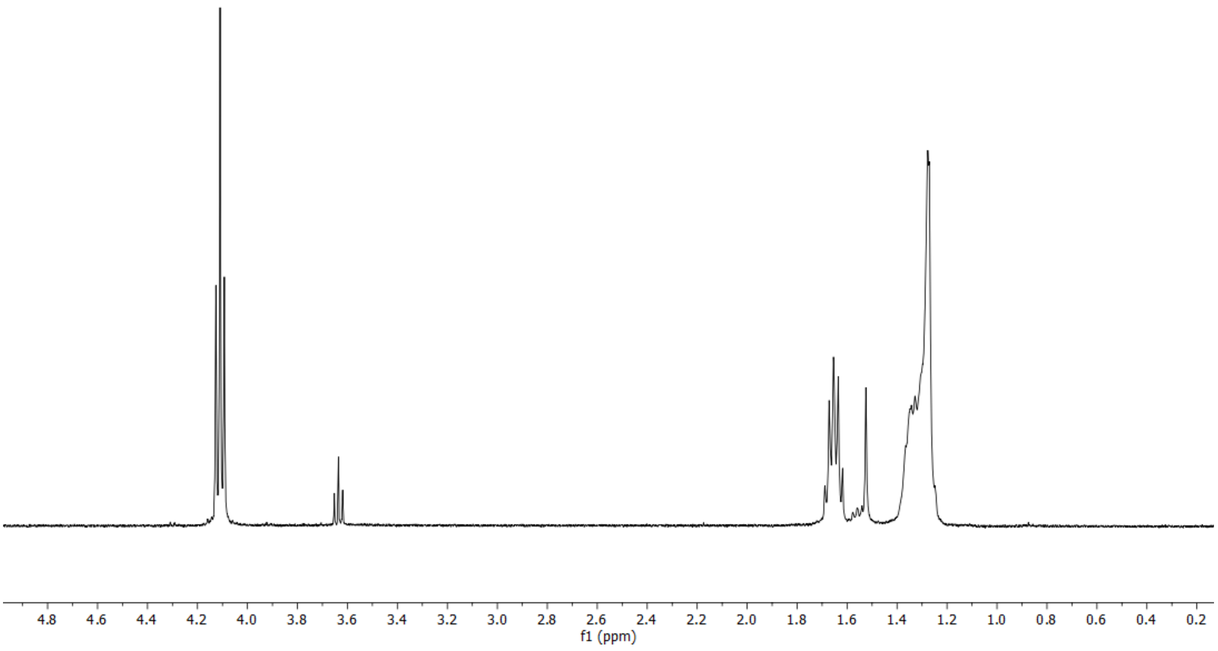

Figure S2. ^{1}H NMR spectrum of the OCD.

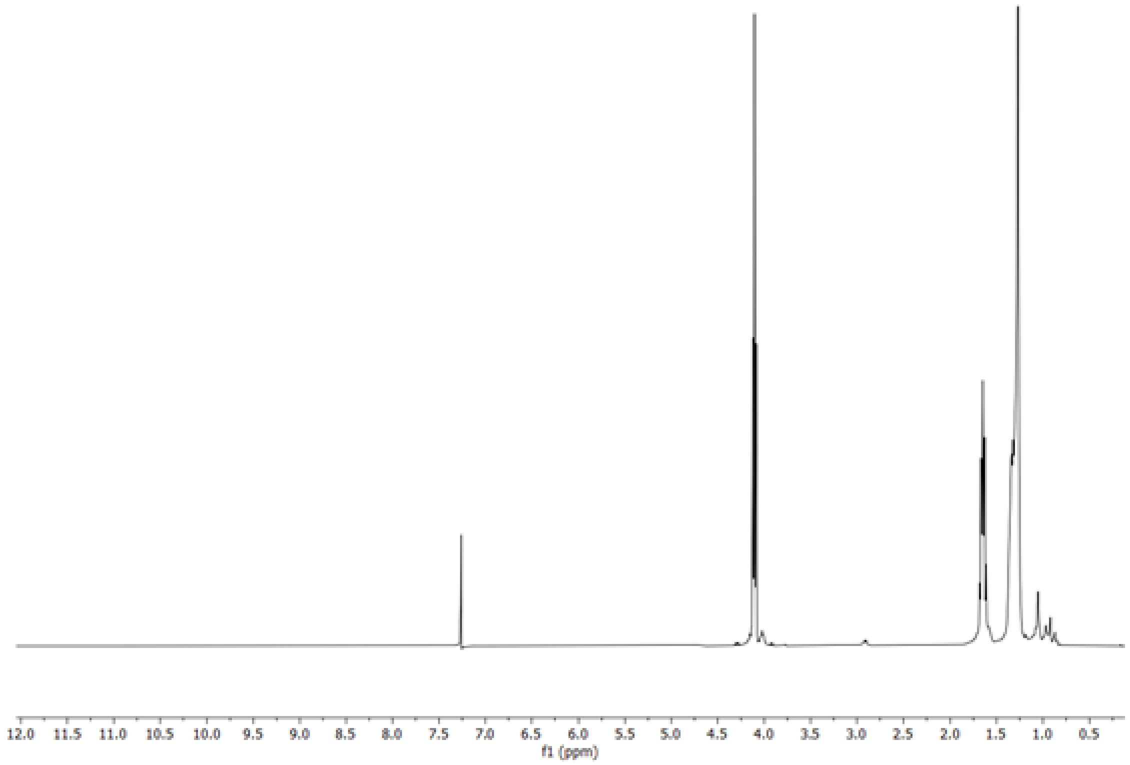

Figure S3. ^{1}H NMR spectrum of the PCUU.

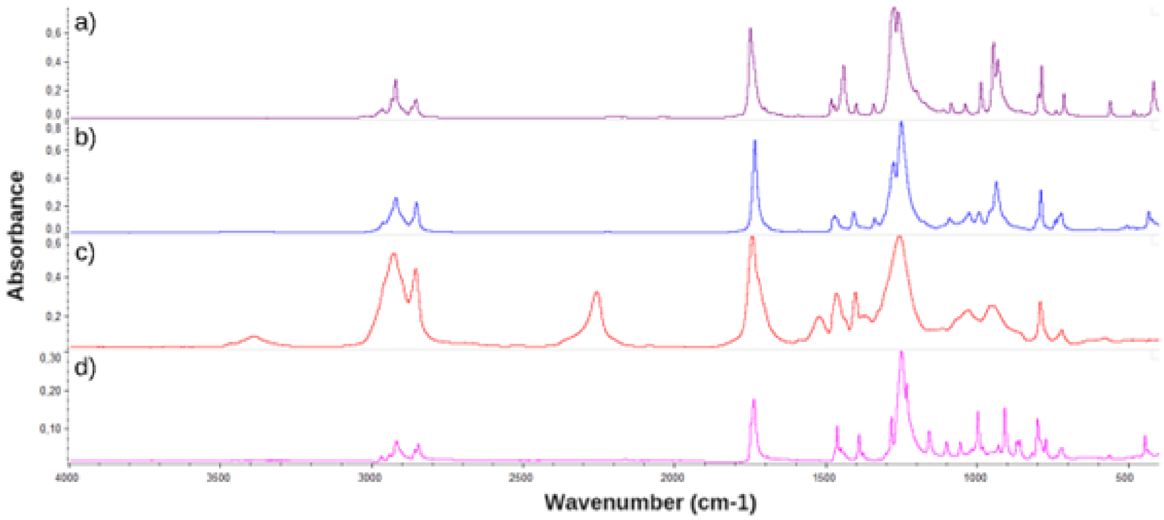

Figure S4. FTIR spectra of a) BCM; b) OCD; c) urethane prepolymer; and d) cured PCUU.

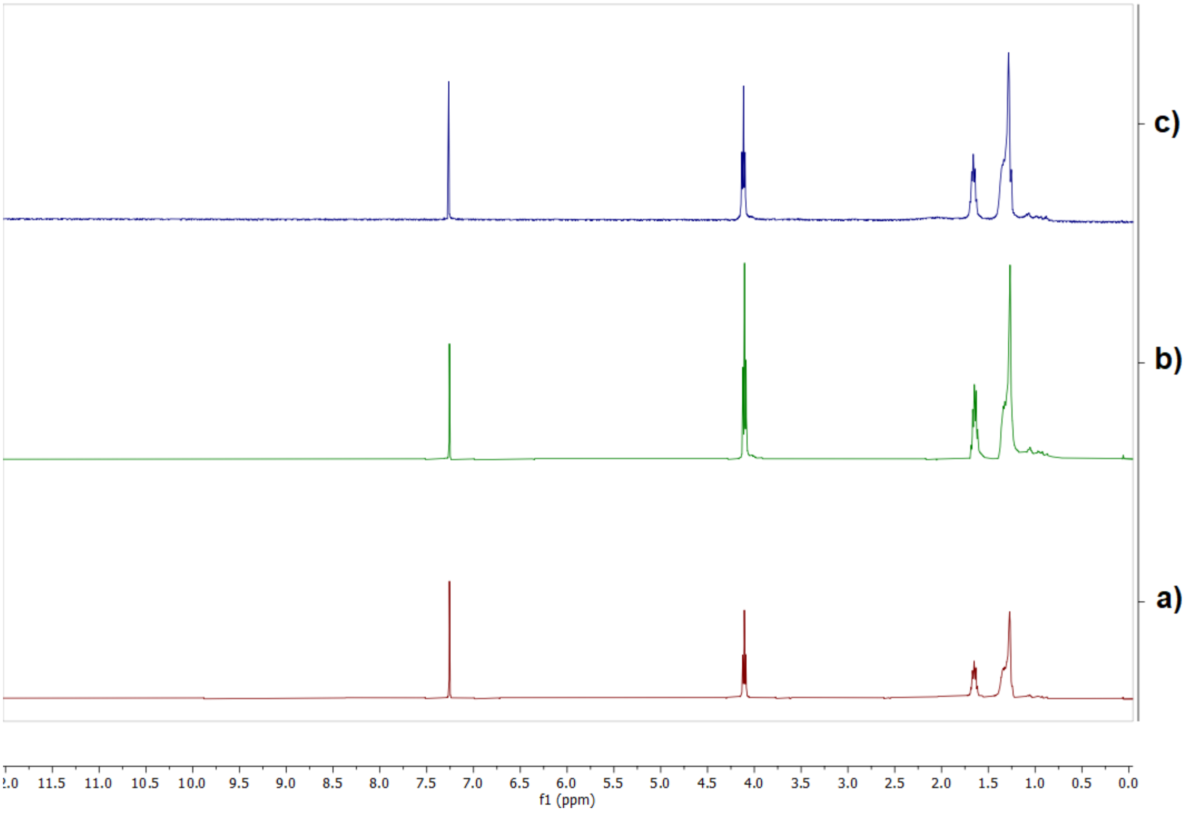

Figure S5. ¹H NMR spectrums of the a) N_3; b) N_4; and c) N_5.

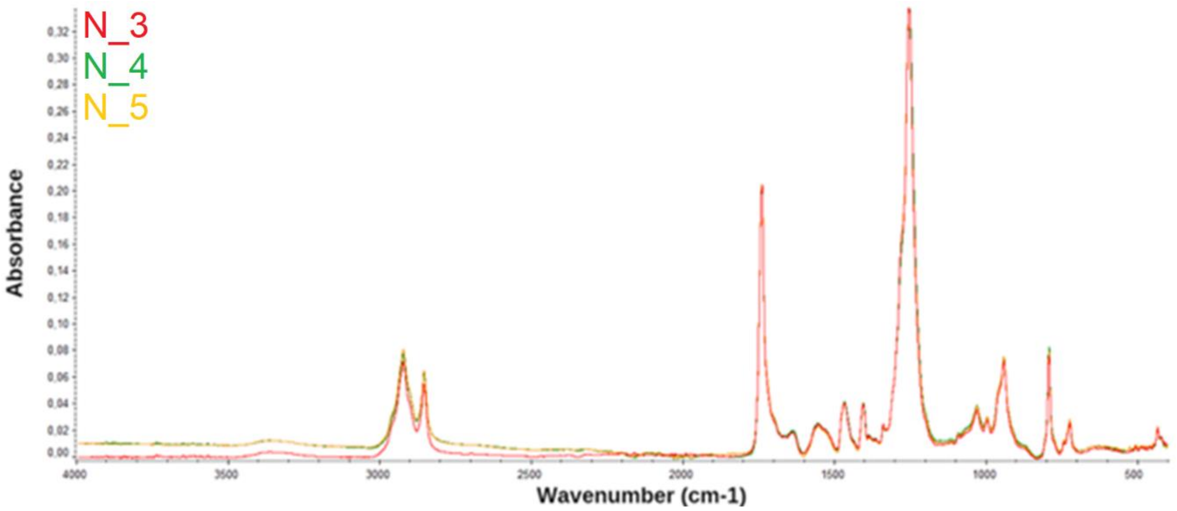

Figure S6. FTIR spectra of N_3 (red line), N_4 (green line); and N_5 (yellow line).

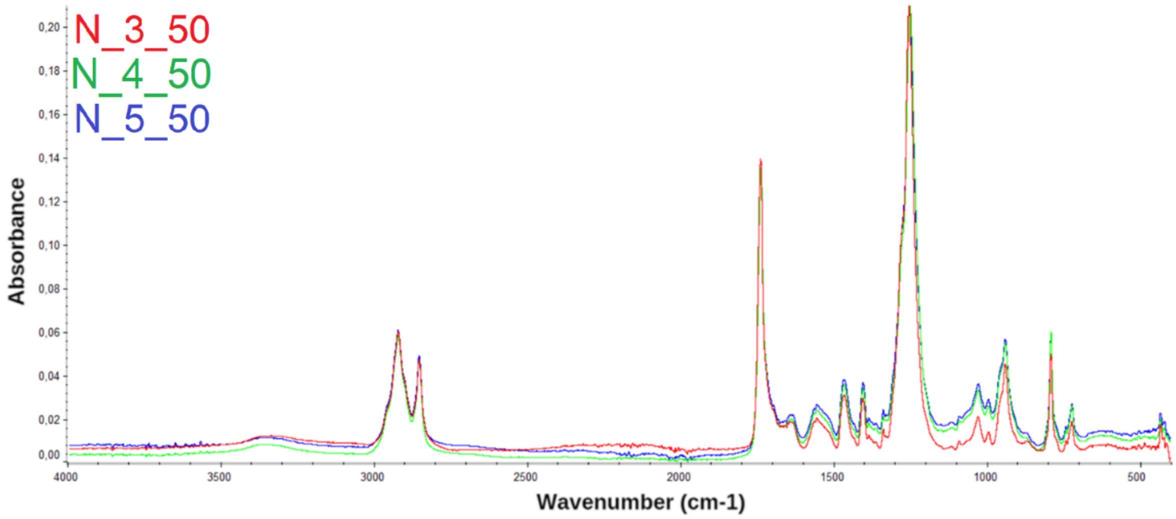

Figure S7. FTIR spectra of N_3_50 (red line), N_4_50 (green line); and N_5_50 (blue line).

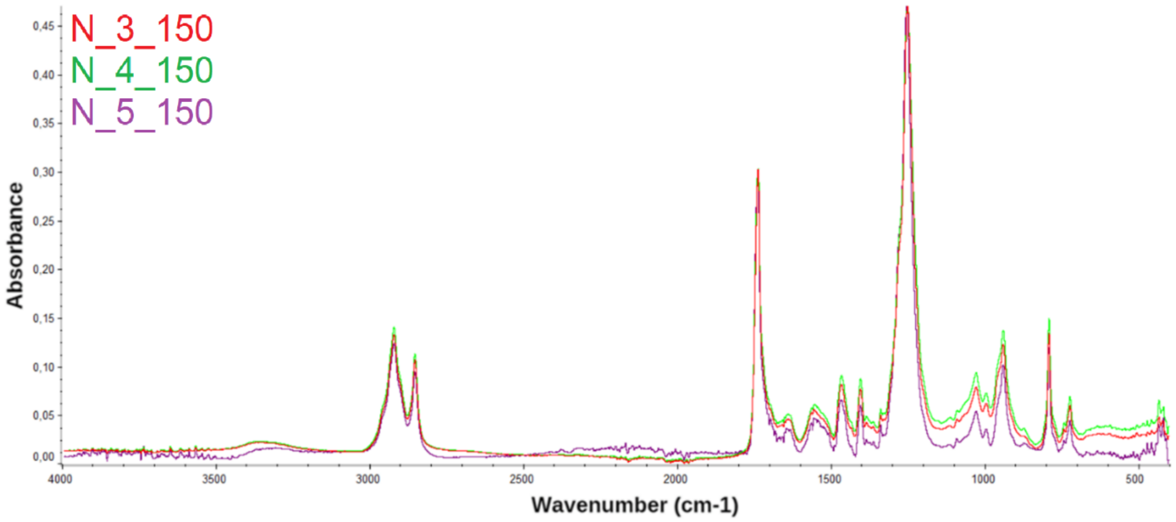

Figure S8. FTIR spectra of N_3_150 (red line), N_4_150 (green line); and N_5_150 (purple line).

Table S2. Porosity and thickness of obtained electrospun PCUU nonwovens and average fibers diameter.

Collector type	Sample name	Average fiber diameter µm	Average porosity %
Plate collector	N_3	0.54 ± 0.14	47.7 ± 2.4
	N_4	0.56 ± 0.21	51.8 ± 1.5
	N_5	0.82 ± 0.12	50.7 ± 1.3
Rotating collector	N_3_50	0.30 ± 0.05	55.6 ± 0.4
	N_4_50	0.54 ± 0.15	51.9 ± 1.8
	N_5_50	0.74 ± 0.18	50.9 ± 2.0
	N_3_150	0.28 ± 0.07	52.0
	N_4_150	0.50 ± 0.09	57.4
	N_5_150	0.63 ± 0.07	58.2

a)

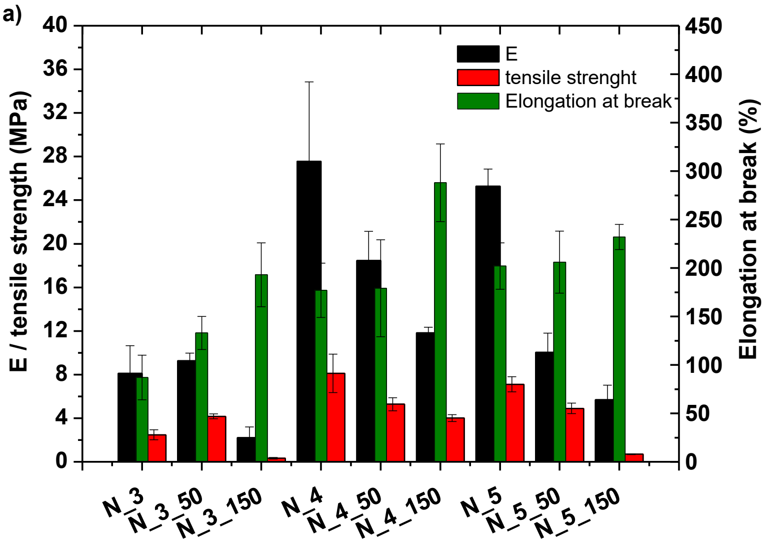

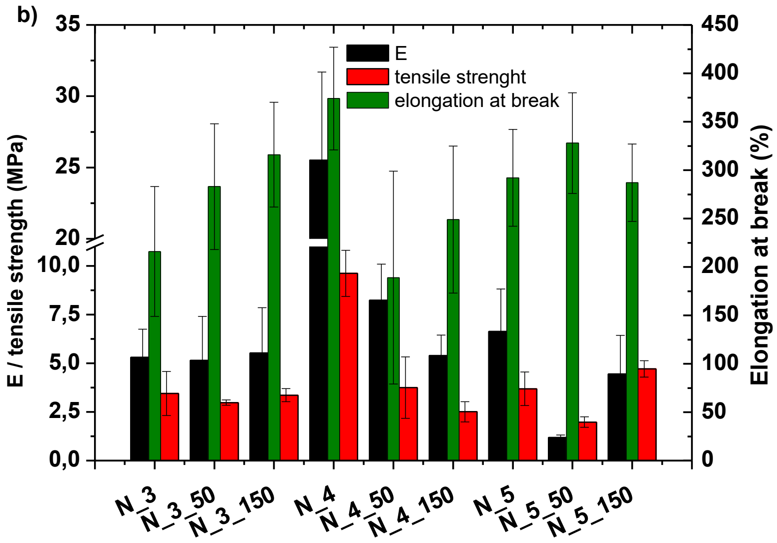

Figure S9. Mechanical properties of samples obtained from solutions with various concentrations and electrospun with various speeds of collector; a) at RT; b) at 37 °C.

Table S3. Thermal properties of PCUU nonwovens based on DSC curves.

Sample	T_g	T_m	ΔH_m	T_{m2}	ΔH_{m2}	T_c	ΔH_c
	1st run			2nd run			
	°C	°C	$J \cdot g^{-1}$	°C	$J \cdot g^{-1}$	°C	$J \cdot g^{-1}$
N_3	-19±1	46±1	26±1	40±1	20±1	-5±1	16±1
N_4	-20±1	46±1	28±1	40±1	18±1	-6±1	16±1
N_5	-20±1	44±1	28±1	41±1	23±1	-6±1	17±1
N_3_50	-	50±1	40±1	40±1	19±1	-8±1	20±1
N_4_50	-	49±1	30±1	40±1	14±1	-9±1	15±1
N_5_50	-	46±1	37±1	39±1	21±1	-8±1	23±1
N_3_150	-	49±1	51±1	45±1	22±1	0±1	22±1
N_4_150	-	49±1	52±1	42±1	23±1	-9±1	23±1
N_5_150	-	48±1	49±1	40±1	21±1	-9±1	23±1

“-“ T_g was not observed in DSC measurements

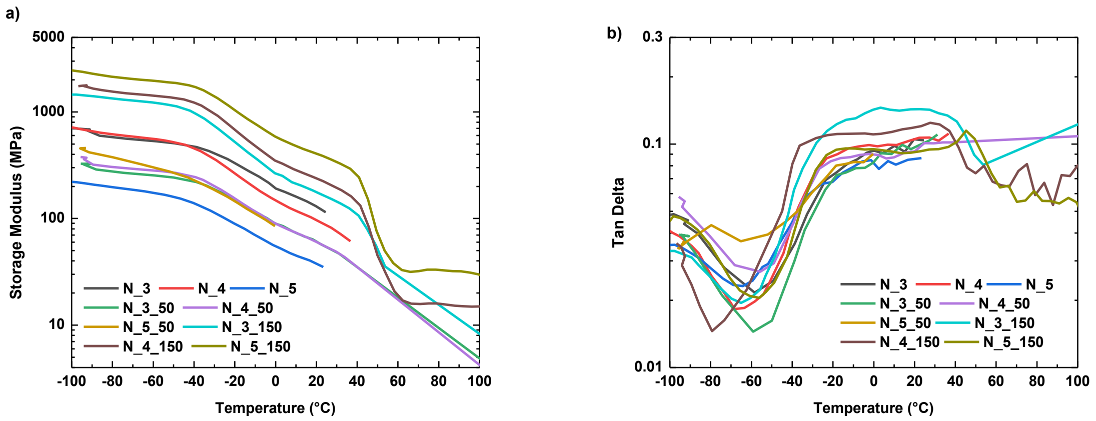

Figure S10. DMTA plots of a) storage modulus E' and b) of Tan Delta as a function of temperature.

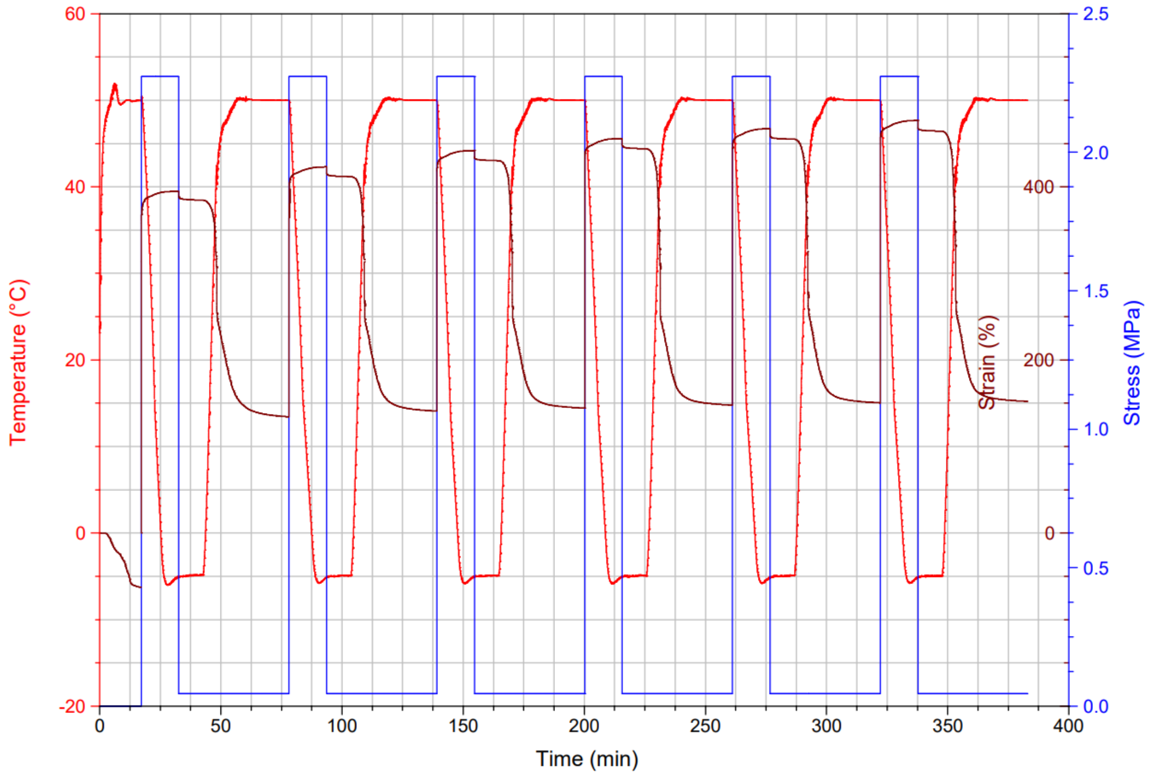

Figure S11. Diagram of one-way shape memory performance of N_3.

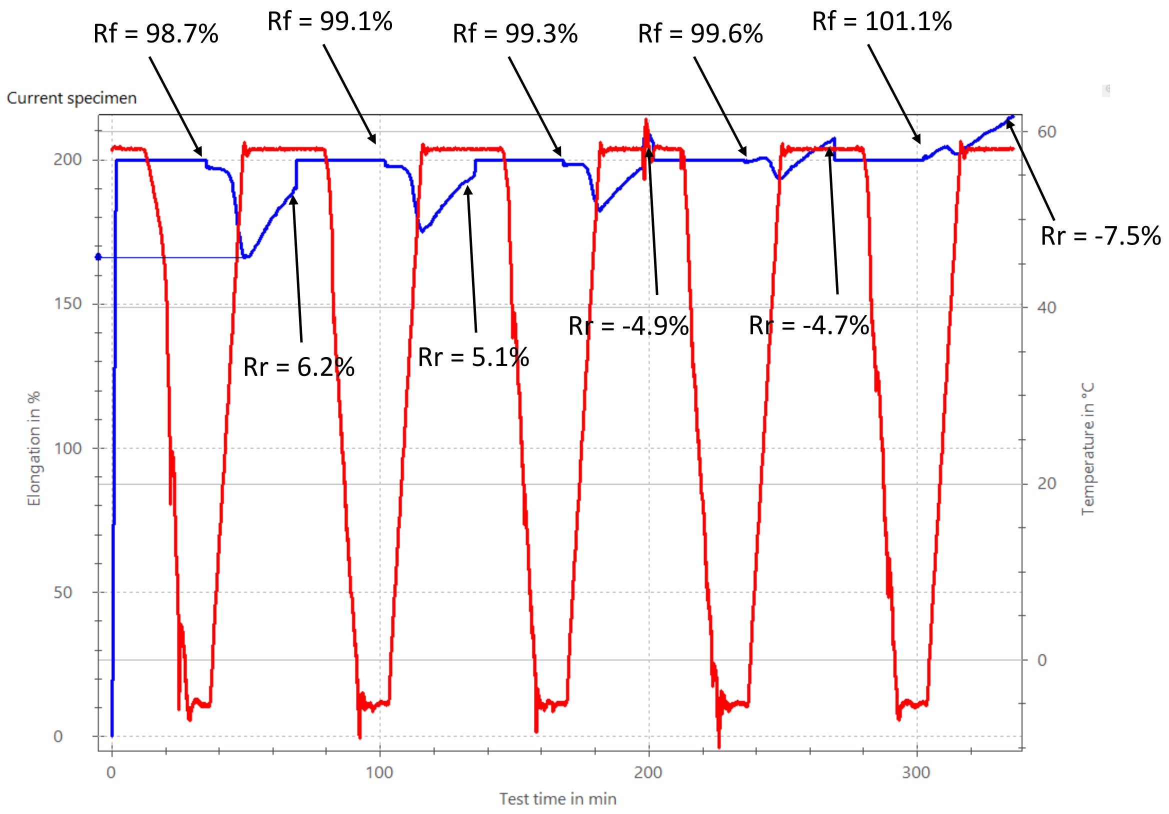

Figure S12. Diagram of one-way shape memory performance of N_4_150 with calculated R_r and R_f in each cycle of the measurement.

Table S4. Fiber diameter based on SEM analysis of PCUU nonwovens incubated in PBS buffer for 0, 7, and 14 days.

**Sample name**	**Incubation time**		
	**0 days**	**7 days**	**14 days**
	**Diameter (μm)**		
N_3_50	0,330±0,116	0,316±0,066	0,345±0,050
N_4_150	0,498±0,094	0,468±0,092	0,520±0,080
N_5_150	0,634±0,066	0,578±0,062	0,531±0,058

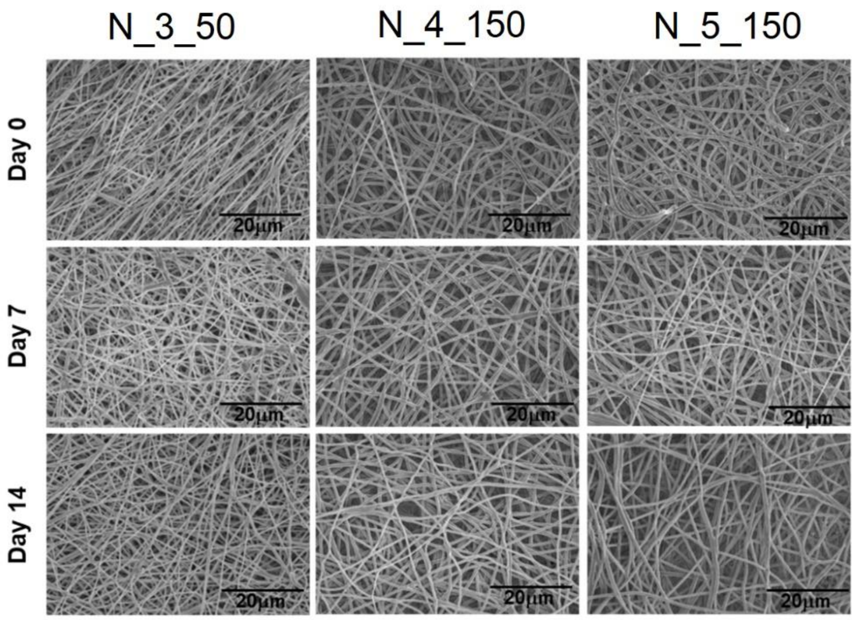

Figure S13. SEM analysis of PCUU nonwovens incubated in PBS buffer for 0, 7, and 14 days.

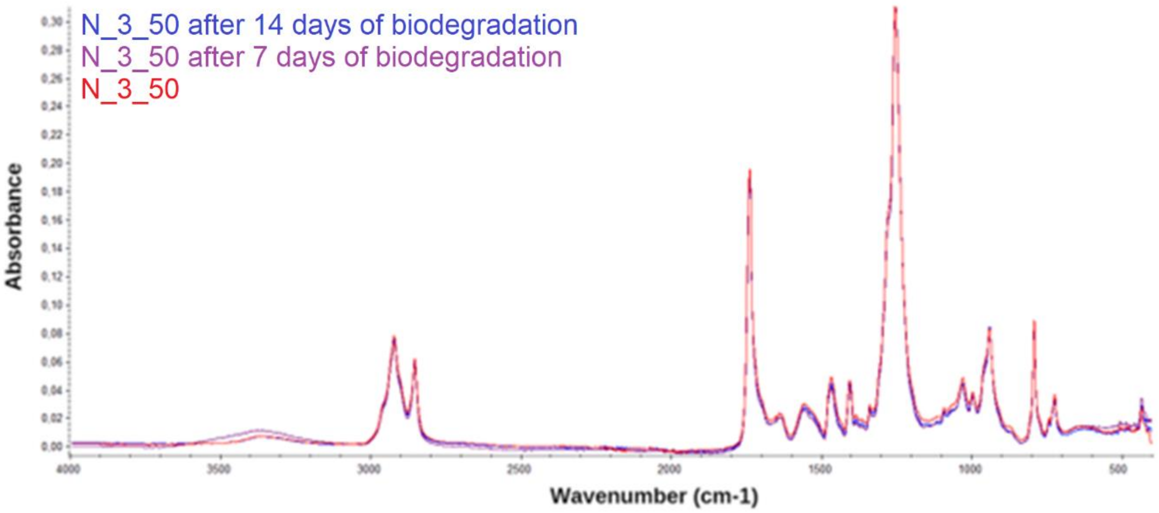

Figure S14. FTIR spectra of N_3_50 sample before and after biodegradation treatment (for 7 and 14 days).

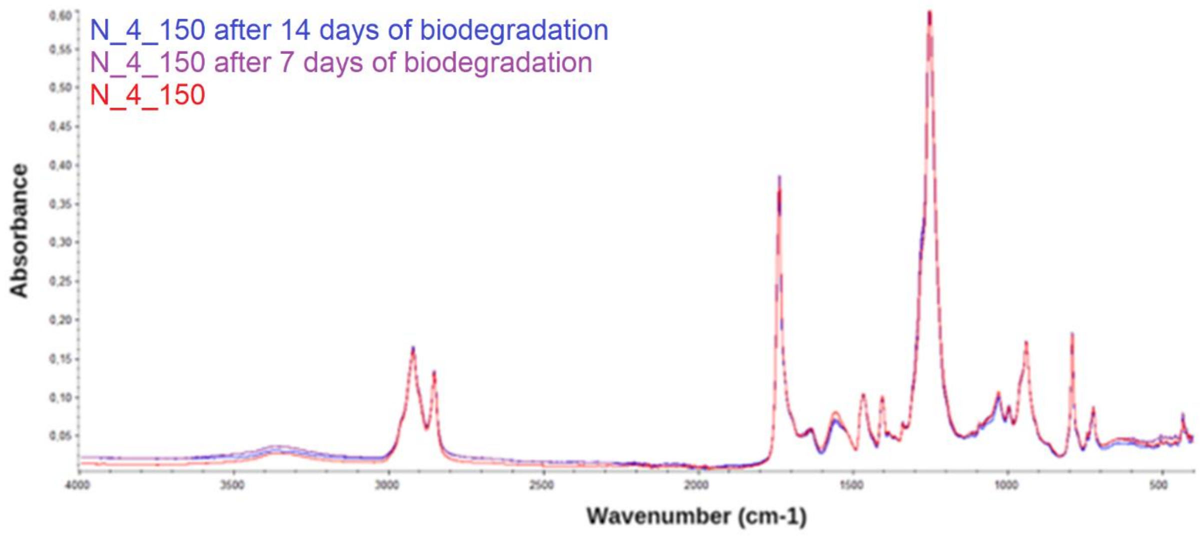

Figure S15. FTIR spectra of N_4_150 sample before and after biodegradation treatment (for 7 and 14 days).

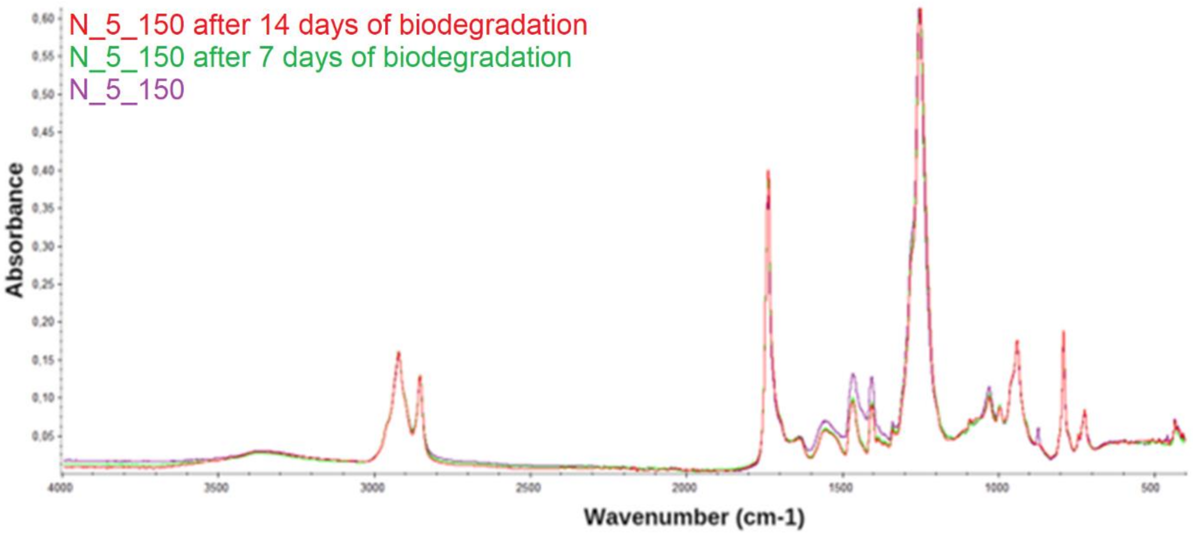

Figure S16. FTIR spectra of N_5_150 sample before and after biodegradation treatment (for 7 and 14 days).
